# Characterization of innate immunity genes in the parasitic nematode *Brugia malayi*

**DOI:** 10.1007/s13199-015-0374-7

**Published:** 2016-01-05

**Authors:** Silvia Libro, Barton E. Slatko, Jeremy M. Foster

**Affiliations:** Genome Biology Division, New England Biolabs, Inc., 240 County Road, Ipswich, MA 01938 USA

**Keywords:** Nematode, *Brugia*, Transcriptomics, *Wolbachia*, Immunity

## Abstract

The filarial nematode *Brugia malayi* is one of the causative agents of lymphatic filariasis, a neglected tropical disease that affects 120 million people worldwide. The limited effectiveness of available anthelmintics and the absence of a vaccine have prompted extensive research on the interaction between *Brugia* and its obligate bacterial endosymbiont, *Wolbachia*. Recent studies suggest that *Wolbachia* is able to manipulate its nematode host immunity but relatively little is known about the immune system of filarial nematodes. Therefore, elucidation of the mechanisms underlying the immune system of *B. malayi* may be useful for understanding how the symbiotic relationship is maintained and help in the identification of new drug targets. In order to characterize the main genetic pathways involved in *B. malayi* immunity, we exposed adult female worms to two bacterial lysates (*Escherichia coli* and *Bacillus amyloliquefaciens*), dsRNA and dsDNA. We performed transcriptome sequencing of worms exposed to each immune elicitor at two different timepoints. Gene expression analysis of untreated and immune-challenged worms was performed to characterize gene expression patterns associated with each type of immune stimulation. Our results indicate that different immune elicitors produced distinct expression patterns in *B. malayi*, with changes in the expression of orthologs of well-characterized *C. elegans* immune pathways such as insulin, TGF-β, and p38 MAPK pathways, as well as C-type lectins and several stress-response genes.

**Electronic supplementary material:**

The online version of this article (doi:10.1007/s13199-015-0374-7) contains supplementary material, which is available to authorized users.

## Introduction

The filarial nematode *Brugia malayi* is one of the causative agents of lymphatic filariasis (elephantiasis), a neglected tropical disease that affects 120 million people in endemic tropical areas. The disease is transmitted to the human host via infected mosquitoes which allow third-stage filarial larvae (L3) to enter the host’s bloodstream. Larvae reach maturity in the lymphatic system and reproduce, generating millions of microfilariae that migrate to the capillaries from where they can be ingested by new mosquitos. In the mosquito, microfilariae develop into first-stage larvae, second-stage larvae and then infective third-stage larvae, and the cycle repeats.

Current treatments such as albendazole and ivermectin rely on mass drug administration (MDA) programs - but they predominantly target larval stages, necessitating a treatment course of up to 10–15 years (Molyneux et al. 2003). Due to this and other limitations of MDA, extensive effort has been devoted to research for alternative treatments. In particular, several projects are currently focusing on understanding the biology of the relationship between *B. malayi* and its obligate endosymbiont, the alpha-proteobacterium *Wolbachia. Wolbachia* is present in intracytoplasmic vacuoles in several filarial nematodes and is required for worm fertility and survival (Foster et al. 2013; Taylor et al. 2005) making it a promising therapeutic target for filariasis control. One example of anti-filarial strategies exploiting this obligate mutualism is the use of antibiotics against *Wolbachia*, such as doxycycline, to cause premature death and permanent sterilization of adult worms.

While the mechanisms of the symbiotic relationship are not fully understood, recent studies suggest that apoptosis and autophagy- two conserved cellular pathways essential for homeostasis and innate immunity- are involved in the regulation of *Wolbachia* titer in filarial nematodes (Landmann et al. 2011; Voronin et al. 2012), suggesting that *Wolbachia* is able to modulate and evade the host immune system in order to survive. In particular, these studies report extensive apoptosis in germline and somatic cells of embryos, microfilariae, and fourth-stage larvae following antibiotic-mediated depletion of *Wolbachia* (Landmann et al. 2011) and show that *Wolbachia* can be recognized and eliminated by autophagy in filarial nematodes and insects (Voronin et al. 2012). Similar to other mutualistic bacterial symbioses, these data are indicative of a cross-talk between *Wolbachia* and its nematode host immune system. Therefore, determining the mechanisms underlying *B. malayi* immunity can provide critical information to understand how the symbiosis with *Wolbachia* is maintained.

So far, few studies have focused on the *B. malayi* immune system and our knowledge is mostly based on sequence similarity with the free-living nematode *Caenorhabditis elegans* (Engelmann and Pujol 2010; Irazoqui et al. 2010). We initiated experiments aimed at the identification of candidate immune-related genes in the filarial nematode *B. malayi*. Live adult females were exposed to four different immune elicitors (dsRNA, dsDNA, Gram-positive and Gram-negative bacterial lysates) for 24 and 36 h. The expression profiles of treated and un-treated worms were compared and the identity of differentially expressed (DE) transcripts was determined based on current genome annotations. Our results indicate that different immune elicitors produced distinct expression patterns in *B. malayi*, with changes in the expression of orthologs of well-characterized *C. elegans* immune pathways such as insulin, transforming growth factor beta (TGF-β), and p38 MAPK pathways, as well as C-type lectins and several stress-response genes.

## Materials and methods

Due to the ex vivo *B. malayi* experimentation and the consequent challenges associated with the use of live bacteria in culture medium (i.e., risk of death by starvation caused by bacterial overgrowth), bacterial lysates were used to simulate bacterial recognition. Additionally, dsRNA and dsDNA were used in order to stimulate the response to exogenous nucleic acids, based on the evidence that small RNAs are key mediators of antiviral immunity in *C. elegans* (Wilkins et al. 2005) and that the genome of *B. malayi* encodes several components of the pathway (Dalzell et al. 2011).

### Preparation of dsRNA

The template for the production of dsRNA was LITMUS 28i (New England Biolabs), a double-stranded cloning/in vitro transcription phagemid vector that has opposing T7 promoters. cDNA was obtained by PCR amplification of a 172 bp region of the vector using Q5® High-Fidelity 2X Master Mix (Cat. #M0492, New England Biolabs) and custom designed primers (IDT): 5-TAA TAC GAC TCA CTA TAG GGC AGA T-3 for the forward primer and 5-TAA TAC GAC TCA CTA TAG GCC TTG ACT AG-3 for the reverse primer. The PCR product was verified by 1 % agarose *gel* electrophoresis and then purified with the QIAquick PCR Purification Kit (Cat. #28104, Qiagen) following the manufacturer’s protocol but with one extra wash with PE buffer. PCR products were subsequently used to synthesize dsRNA using the HiScribe T7 Quick High Yield RNA Synthesis Kit (Cat. #E2040S, New England Biolabs) followed by DNase I (Cat. # AM1907, Ambion) treatment according to the protocol. Concentration and size of dsRNA were assessed respectively with the Qubit® RNA HS Assay Kit (Cat. #Q32852, Life Technologies) on a Qubit® Fluorometer and with the RNA 6000 Nano Kit (Cat. #5067-1511, Agilent Technologies) on a Bioanalyzer 2100.

### Preparation of dsDNA

Unmethylated dsDNA was obtained from Lambda vector DNA (Cat. #N3013, New England Biolabs). A 3 ml stock of 2.2 mg/ml Lambda DNA was diluted five-fold in RNAse free water and sheared to approximately 150 bp using AFA Covaris microtubes (Cat. #520045, Covaris) on a Covaris S2 sonicator. Size and concentration of dsDNA were assessed by electrophoresis on 1 % agarose gels and confirmed with the Qubit® dsDNA HS (High Sensitivity) Assay Kit (Cat. #Q32851, Life technologies) and the Agilent DNA 1000 Kit (Cat. #5067-1504, Agilent Technologies).

### Bacterial lysates

Lysates of *Escherichia coli* and *Bacillus amyloliquefaciens* were prepared by centrifuging bacterial cultures in a Sharples continuous-flow centrifuge at 17,000 rpm. Cells were then frozen to −80 °C, thawed overnight at 4 °C and diluted with water (1:3) to a final concentration of 33 g per liter prior to homogenization in a Microfluidizer^®^ (Microfluidics) at 20,000 psi. Lysates were stored at −20 °C, and before being used were thawed at 4 °C and warmed to 37 °C.

### Medium preparation

A stock of 5X culture medium was prepared by adding 86 mL of sterile water, 9.6 mL of 100X antibiotic-antimycotic solution containing 10,000 units penicillin, 10 mg streptomycin and 25 μg Amphotericin B per ml (Cat. #A-5955, Sigma-Aldrich, St Louis, MO, USA) plus 145 mg of L-Glutamine, 1.92 g of NaHCO3 and 0.00096 g of folic acid to 96 mL of 10X of RPMI-1640 cell culture medium (Cat. #R1145, Sigma-Aldrich, St Louis, MO, USA). A second stock without the antibiotic was made by adding 89 mL of sterile water, 1 mL of 100X glutamine (Cat. #25030-081, Thermo-Fisher Scientific), 0.38 g of NaHCO3 and 0.0002 g of folic acid to 100 mL of 10X of RPMI-1640 cell culture medium. Both stocks were filtered in a Nalgene filtration bottle (Nalgene, Rochester, NY, USA) in a laminar flow hood, stored at 4 °C and pre-warmed to 37 °C before use.

### Experiment set up

All equipment was sprayed with 70 % ethanol before use under a laminar flow hood. Two sterile 12-well cell culture plates were set up as follows: each well of the first plate received 200 μl of 5X medium with antibiotic-antimycotic solution. Wells 1 to 4 received 200 μl of dsRNA and 600 μl of sterile water (2.5 uM dsRNA final concentration); wells 5 to 8 received 800 μl of sheared DNA (2.5 uM dsDNA final concentration) and wells 9 to 12 received 800 μl of sterile water (control plus antibiotic). In the second plate, wells 1 to 8 received 200 μl of 5X medium with antibiotic-antimycotic solution, 300 μl of sterile water and 200 μl of *B. amyloliquefaciens* (*B.a*.; wells 1 to 4) or *E. coli* lysate (*E.c*.; wells 5 to 8). Wells 9 to 12 received 200 μl of 5X medium and 800 μl of sterile water (control without antibiotic).

Adult *B. malayi* females were obtained from the NIH/NIAID Filariasis Research Reagent Resource (FR3) Center. After 2 h of acclimation in 1X RPMI-1640 cell culture medium with L-glutamine (Invitrogen, Carlsbad, CA, USA) at room temperature, two worms were transferred to each well of the two 12-well cell culture plates using a curved pick. The plates were cultured at 37 °C in 5 % CO_2_ under a laminar flow hood. The medium along with each treatment was replaced every 12 h. Worms were inspected approximately every hour during daytime for the duration of the treatment to assess viability and motility, and each worm’s behavior was recorded with scores ranging from 3 (normal activity) to 0 (no motility) as described by Keiser (Keiser 2010). Two replicates (i.e., wells) of each treatment were collected at each of the two time points (24 and 36 h) and placed in 1.5 mL DNA LoBind Tubes (Eppendorf, Hamburg, Germany) and stored at −80. Exceptions were worms exposed to dsDNA, collected after 12 and 16 h, and to *E. coli* lysate, collected after 20 and 22 h, due to a rapid decrease in motility compared to the other treatments.

### RNA extraction and library preparation

Total RNA was extracted using a modified organic extraction protocol using Trizol (Ambion). Each pair of worms was homogenized in 300 μl Trizol using disposable plastic pestles in the original collection tube. A further 200 μl of Trizol was used to rinse the pestle in the tube, then 10 μl of proteinase K (Cat. #P8107, New England Biolabs) was added, followed by 30 min of incubation at 55 °C. To each tube 270 μl of chloroform was added and tubes were incubated for 3 min at room temperature. Samples were then transferred into Phase Lock Gel Heavy Tubes (Cat. #0032005152, Eppendorf) with 65 μl of fresh Trizol. Samples were centrifuged at 12,000 X g for 15 min at 4 °C and the upper aqueous phase was transferred to a DNA LoBind tube. After addition of 5 μL of linear acrylamide (Cat. #E6103, New England Biolabs) and 50 μl of sodium acetate pH 5.2, RNA was recovered by precipitation with isopropanol, washed with 70 % ethanol three times and resuspended in 50 μl of RNAse free water.

Extracted RNA was then treated with DNase I (Cat. #AM1907, Ambion) and purified using RNA Clean & Concentrator^™^-5 according to the manufacturer’s protocol (Cat. #R1013, Zymo Research). The integrity, purity and concentration of all RNA samples were assessed on an RNA nano chip using an Agilent Bioanalyzer 2100. *B. malayi* mRNA was isolated using theNEBNext Poly(A) mRNA Magnetic Isolation Module (Cat. #E7490, New England Biolabs) and transcriptomic libraries were prepared using the NEBNext^®^ Ultra™ RNA Library Prep Kit for Illumina (Cat. #E7530, New England Biolabs) and the NEBNext^®^ Multiplex Oligos for Illumina (Index Primers 1–12) (Cat. # E7335, New England Biolabs) following the kit protocol. Library quality and concentration was assessed using a DNA high sensitivity chip on a Bioanalyzer 2100.

### Transcriptome sequencing and analysis

Due to low yield, two libraries were not included in the further analysis: one obtained from worms exposed to medium without antibiotic for 36 h, the other from worms exposed to dsDNA for 16 h. The remaining 22 cDNA libraries (two replicates per treatment), were multiplexed into two pooled samples and sequenced using an Illumina MiSeq platform (Illumina, San Diego, CA, USA) with150 bp paired-end reads. The resulting FASTQ files were imported on a local instance of Galaxy (Blankenberg et al. 2010; Giardine et al. 2005; Goecks et al. 2010) for quality filtering and adaptor trimming. Reads were aligned to the *B. malayi* genome (version: WS247) using TopHat (Galaxy Tool Version 1.5.0) (Trapnell et al. 2009) with default parameters and assembled into transcripts with Cufflinks (Galaxy Tool Version 0.0.7) with quartile normalization and multi-read correct options. All the resulting Cufflinks assemblies were then merged with Cuffmerge (Galaxy Tool Version 0.0.6) into a single transcript model. To calculate gene expression levels and statistical significance of expression differences, pairwise comparisons between treatments and controls were performed in Cuffdiff (Galaxy Tool Version 0.0.7) with geometric normalization and false discovery rate (FDR) of 0.01. The expression level for each transcript was expressed as log2 fold change of Fragments Per Kilobase of transcript per Million mapped reads (FPKM)-normalized count data.

Transcripts were annotated using a combination of existing annotations retrieved from WormBase and Uniprot databases. Clusters of genes exhibiting significant functional annotation enrichment were identified using the Functional Annotation Clustering tool in DAVID (Database for Annotation, Visualization, and Integrated discovery) (Huang et al. 2008). Among the annotated DE transcripts, putative immune-related genes were identified by manual curation based on literature searches.

## Results and discussion

Transcriptome sequencing of the 22 libraries included in this study yielded a total of 4.2 × 10^8^ read pairs, with an average of 1.9 × 10^7^ (±1.0 × 10^7^) reads per samples. An average of 1.3 × 10^7^ (±8.0 × 10^6^) pairs mapped to the *B. malayi* reference genome, of which 82.7 % (±12.7 %) were concordant read pairs (See ESM 1).

To account for the effects of the presence of antibiotic/antimycotic mixture in the medium, gene expression comparisons between controls with and without the antibiotic/antimycotic mixture were assessed. Transcriptome comparisons of worms exposed to medium with (C + A) and without antibiotic (C-A) yielded 70 (21 annotated) DE genes after 24 h incubation and 84 (24 annotated) after 36 h. These transcripts did not show any significant functional enrichment based on the DAVID Functional Annotation Clustering (Fisher exact P-value <0.1) and did not include any known immune mediator (See ESM 2). This was not surprising, as the antibiotics added to the medium are routinely used in the preparation of culture medium to prevent the growth of undesired microorganisms and do not affect *Wolbachia* (Landmann et al. 2012; Marcellino et al. 2012)

**Fig. 1:**
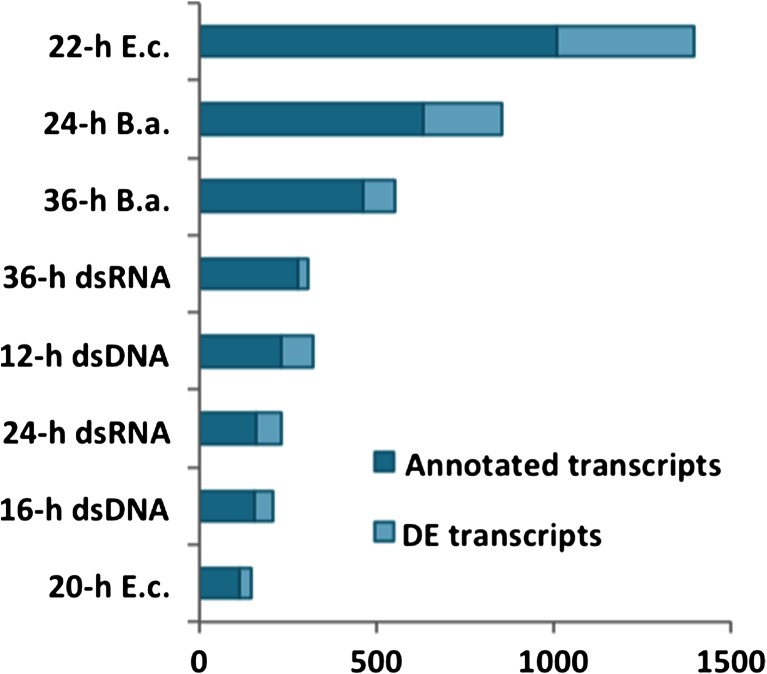
Differentially expressed (DE) genes between treated worms and controls. Bar charts depicting the number of differentially expressed (adj-p < 0.1) transcripts between treated worms and controls. Worms were exposed to the following treatments: dsRNA for 24 and 36 h (24-h and 36-h dsRNA), dsDNA for 12 and 16 h (12- and 16-h dsDNA), *E. coli* lysate for 20 and 22 h (20- and 22-h *E. c*.) and *B. amyloliquefaciens* for 24 and 36 h (24- and 36-h *B. a*.). Expression changes were relative to 24-h C + A (medium plus antibiotic) for all treatments except for 36-h dsRNA and 36-h *B. a*., which were compared to 36-h C + A

The number of DE transcripts between treatments and controls is shown in Fig. 1. Overall, the two bacterial treatments (22-h *E. c*. and 24-h *B. a*.) induced the strongest expression changes relative to 24-h C + A with 1008 (390 annotated) and 632 (220 annotated) DE transcripts respectively. This was not entirely unexpected, as the addition of bacterial lysates to the culture resulted in increased turbidity and color change of the medium. This likely contributed to the decline in motility in *E. coli* treated worms at 20 and 22 h post-treatment. Interestingly, worms exposed to *B. amyloliquefaciens* lysate did not exhibit the same alterations in motility. Exposure for 36 h to *B. amyloliquefaciens* lysate (36-h *B. a*.) and to dsRNA (36-h dsRNA) yielded 462 and 279 DE transcripts (89 and 28 annotated) compared to 36-h C + A, while comparisons between the dsDNA treatments (12 and 16-h dsDNA) and 24-h C + A yielded 233 and 154 transcripts (89 and 55 annotated).

In general, the top DAVID functional clusters were represented by EGF-like domains, transmembrane proteins, cell adhesion, ion binding, peptidases/metallopeptidase and structural proteins. EGF-like domain was the most overrepresented category in worms exposed to 12-h dsDNA, 24-h dsRNA, 22-h *E. c*. and 24-h dsRNA, while DNA binding, ion binding, structural proteins and transmembrane proteins were enriched in worms exposed to 16-h dsDNA, 36-hs dsRNA, 20-h *E. c*. and 36-h *B. a*. respectively (see EMS2). Two functional clusters were only found in the transcriptome of worms exposed to bacterial treatments, and were represented by immunoglobulin-like (Ig-like), and Leucine rich repeats (LRRs), both candidate immune-related gene families.

### Candidate immune genes

**Fig. 2:**
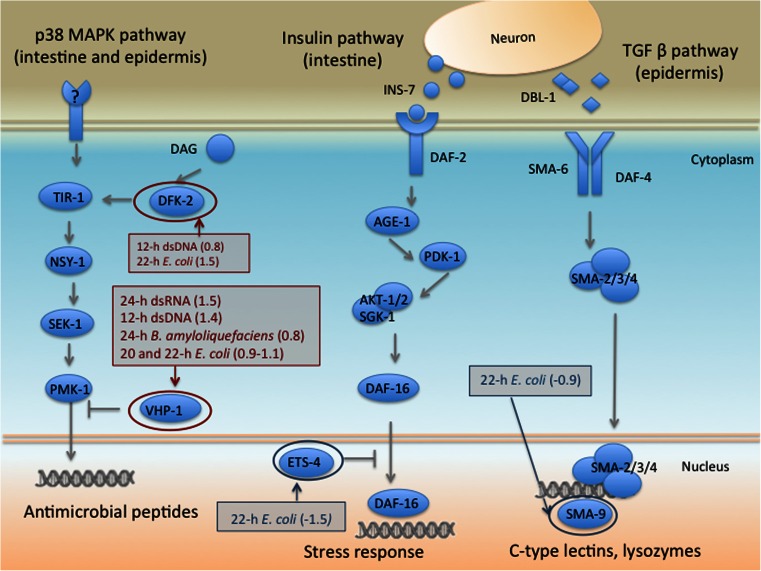
Immune signaling in *C. elegans* host defense. In *C. elegans*, the TGF-β pathway regulates body morphology and immunity, the p38 MAPK pathway induces synthesis of C-type lectins and antimicrobials in response to bacterial pathogens and the Daf2-Daf16 (aka FOXO) insulin pathway is activated by stress and bacterial infection. *Red* and *blue circles* indicate components of the pathways that exhibited respectively up- or down-regulation in immune-challenged *B. malayi* females

In order to identify genes with putative immune functions in *B. malayi*, the annotations of the DE transcripts were manually curated with particular attention to putative immune-related domains and structures, including C-type lectins, LRRs and Ig-like domain-containing proteins, as well as other known immune regulators in *C. elegans*. These included components of three key immune signaling pathways in *C. elegans*, p38 MAPK pathway, the insulin pathway and the TGF-β pathway (Fig. 2). Based on our data, immune challenge of *B. malayi* triggered alterations in two transcripts with opposite activity on the intestinal p38 MAPK pathway- protein kinase D (Dfk-2) and tyrosine-protein phosphatase Vhp-1. Dfk-2 was up-regulated in 12-h DNA and 22-h *E. c*. (0.8 and 1.5 *log*_*2*_ fold change, respectively). In *C. elegans*, Dfk-2 is known to stimulate synthesis of inducible defenses such as C-type lectins and antimicrobials (Hoeckendorf et al. 2012). The expression of the other DE transcript in the pathway, Vhp-1, increased in most treatments (1.5 in 24-h dsRNA, 1.4 in 12-h dsDNA, 0.9 in 20-h *E. c*., 1.1 in 22-h *E. c*. and 0.8 in 24-h *B. a*.). Vhp-1 is an integrator of the stress response provoked by heavy metals and by infection (Kim et al. 2004; Mizuno et al. 2004) and acts as inhibitor of the p38 MAPK pathway.

**Fig. 3:**
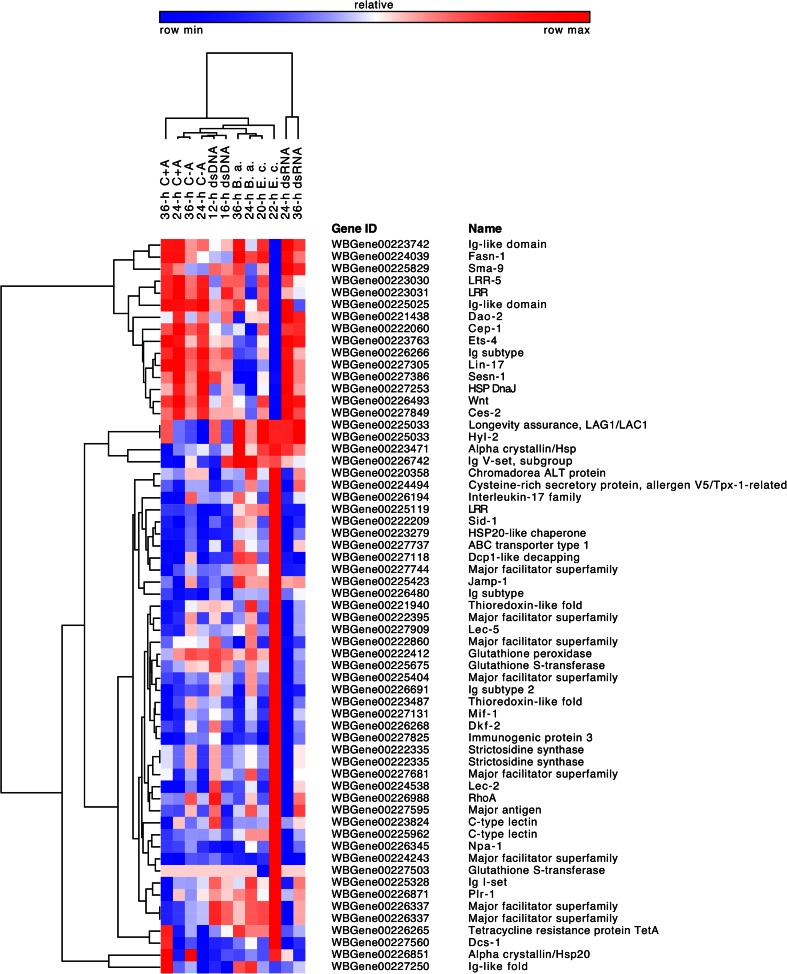
Clustered heatmap of candidate immune-related genes included in this study. Hierarchical clustering of samples and genes was performed using Pearson correlation metrics. *Red* color corresponds to up-regulated genes, *blue* indicates down-regulation. The expression level for each transcript was calculated as log2 fold change of Fragments Per Kilobase of transcript per Million mapped reads (FPKM)-normalized count data. Only genes with a putative immune function are included in the heatmap

A second component of *C. elegans* immunity is the Transforming Growth Factor β (TGF-β) pathway, involved in regulation of body morphology and innate immunity in the epidermis (Roberts et al. 2010). The pathway is activated by the binding of Dbl-1, a TGF-β homolog produced by neurons, to a transmembrane receptor which - similar to the p38 MAPK pathway - stimulates the expression of antibacterial genes, including lectins, lysozymes and antimicrobials like saposins (Zugasti and Ewbank 2009). Worms exposed to *E. coli* lysate for 22 h exhibited down-regulation (−0.9) of Sma-9, a zinc finger transcription factor that acts downstream of Dbl-1 and is thought to confer specific responses to the TGF-β pathway activation (Liang et al. 2003). Sma-9 does not seem to be involved in the production of antimicrobials in *C. elegans*, but rather in the regulation of body size and development (Liang et al. 2003). However, microarray analysis of loss of function mutant worms indicates that C-type lectins and lysozymes are among the targets of Sma-9 (Liang et al. 2007). Interestingly, while the study cited above suggests that Sma-9 is a transcriptional activator of C-type lectin genes, our data showed increased expression of C-type lectins in 22-h *E. c*. treated worms (Fig. 3).

Activation of intestinal insulin signaling pathway by stress or pathogen infection regulates the expression of innate immune genes (Garsin et al. 2003; Singh and Aballay 2009) via nuclear translocation and transcriptional activation of the FOXO transcription factor Daf-16 (Casper et al. 2014). Although no mediator of the insulin pathway was DE in treated worms, the transcription factor Ets-4, that acts as inhibitor of Daf-16, was down-regulated in 22-h *E. c*. (−1.5). A downstream mediator of the pathway - the DB module protein Dao-2 (Yu and Larsen 2001) - was also down-regulated in worms exposed to 22-h *E. c* (−1.9) but was up-regulated by 36-h dsRNA (1.3). Dao-2 expression is regulated by Daf-16 and its activity has been associated with oxidative stress response in *C. elegans* (Shin et al. 2011).

### Small-RNA pathway

The small RNA (smRNA) signaling pathway is a conserved mechanism of antiviral defense (siRNA) and post-transcriptional gene regulation (miRNA) via mRNA degradation, translation repression or DNA methylation. smRNAs are generated from either exogenous (during antiviral defense) or genome-encoded (during post-transcriptional regulation) double-stranded RNA that is processed into 21–26 nt fragments by the enzyme Dicer. The mature fragments are then incorporated into the RNA-induced silencing complex (RISC) and then bind to complementary target mRNA sequences promoting their degradation by Argonaute proteins. In *C. elegans*, the role of miRNAs during stress and pathogen infections has already been reported (Ren and Ambros 2015). While little is known about whether a similar relationship between miRNAs and immunity exists in *B. malayi*, Dalzell et al. (Dalzell et al. 2011) highlighted that the genome of *B. malayi* encodes several components of the pathway. Interestingly, only worms exposed to 22-h *E. c*. exhibited signs of increased smRNA signaling, represented by the up-regulation of the dsRNA transporter Sid-1 (1.1) and of two transcripts encoding decapping enzymes involved in the turnover of miRNAs (Bosse et al. 2013): Decapping scavenger enzyme 1 (Dcs-1, 1.1) and Dcp1-like decapping enzyme (Dcp-1, 0.7). In *C. elegans*, Sid-1 enables dsRNA uptake from the culture medium (Shih and Hunter 2011), therefore it is possible that its overexpression indicates internalization of bacterial dsRNA. Argonaute proteins and other components of the small RNA pathways, although present in our dataset, did not show significant expression changes.

### Wnt pathway

Wnt signaling is a ubiquitous pathway involved in cell fate determination and development but also in immune modulation in several systems, including *C. elegans* (Hoeckendorf et al. 2012). In this experiment, Wnt signaling activity was perturbed as indicated by the DE of two activators of the pathway, abnormal cell lineage (Lin-17), a frizzled receptor that initiates the signaling pathway (Irazoqui et al. 2008), and its ligand Wnt. Lin-17 was down-regulated in 22-h *E. c*. (−1.4), in 24- and in 36-h *B. a*. (−1.3 and −1.2), while Wnt was down-regulated in 22-h *E. c*. (−1.5) and 24-h *B. a*. (−1.0). Consistently, worms exposed to 22-h *E. c*. exhibited up-regulation of one inhibitor of the pathway, Cell polarity defective (Plr-1; 0.8). Plr-1, also up-regulated in 36-h dsRNA (0.7) and in 36-h *B. a*. (1.0), is a transmembrane RING finger protein that acts by down-regulating cell surface levels of frizzled receptors including Lin-17 (Moffat et al. 2014). A fourth component of the pathway that acts downstream of frizzled to regulate cell adhesion and cytoskeletal rearrangements, RAS-like GTP-binding protein RhoA, was also up-regulated in the 22-h *E. c*. treatment (1.1). Taken together, these data suggest that exposure to bacterial lysates and in particular 22-h exposure to *E. coli*, may have an inhibitory effect on the pathway.

### Apoptosis

Apoptosis has been extensively studied in *C. elegans*, where it is required for normal development and homeostasis (reviewed in (Lettre and Hengartner 2006)). In *C. elegans* two Ces (Cell death specification) genes are involved in decision making (Ellis and Horvitz 1991): Ces-2 is a basic leucine zipper (bZip) transcription factor that regulates the activity of Ces-1, a Snail family zinc finger transcription factor. Ces-1 blocks cell death by inhibiting the activity of Egl-1, that together with Ced-3, Ced-4 and Ced-9 represents the downstream execution genes (Metzstein and Horvitz 1999). Apoptosis is initiated by the transcriptional induction of Egl-1 that binds to Ced-9 to release it from Ced-4 (Conradt and Horvitz, 1998; del Peso et al., 2000). After being released from Ced-9, Ced-4 can translocate to the nuclear membrane (Chen et al., 2000) and bind to Ced-3 thereby activating it (Yang et al. 1998). The last part of the apoptotic machinery is represented by Ced-1, Ced-2, Ced-5, Ced-6, Ced-7, and Ced-10, involved in the phagocytic engulfment of cell corpses during programmed cell death (Wu and Horvitz 1998).

In this experiment, two apoptosis mediators were down-regulated: Ces-2, was down-regulated after exposing *B. malayi* to *E. coli* for 22 h (−1.3), while *C. elegans* p53-like–1 (Cep-1) was down-regulated in 22-h *E. c*. (−3.1), in 24-h *B. a*. (−2.9) and in 16-h dsDNA (−1.9) treatments. Cep-1 is a homolog of the mammalian p53 tumor suppressor protein (Derry et al. 2001) and regulates UVC-induced apoptosis and autophagy by activating the transcription of Egl-1 (Stergiou et al. 2007; Tasdemir et al. 2008). In the absence of stress, p53 is maintained at low levels via proteosome-mediated degradation, while upon stress, p53 levels stabilize and activate stress response programs including cell death (Baruah et al. 2014). The link between apoptosis and stress resistance in *C. elegans* has been highlighted even further by mutational studies showing that Ced genes, in addition to killing and removing target cells, can also influence environmental stress resistance (Judy et al. 2013). Taken together, these data indicate that the exposure of *B. malayi* to bacterial lysates resulted in down-regulation of several transcripts with a critical role in stress and antibacterial response. While the reason for this down-regulation is not clear, it is possible that it is the result of complex feedback regulatory mechanisms that act to prevent damage resulting from hyperactivation of the immune system. Similar to other organisms, in *C. elegans* trade-offs exist between immunity and cell metabolism, and activation of immune responses may result in suppression of normal homeostasis to maximize survival during bacterial infection (Ren et al. 2009).

### Other immune genes

Other immune-related categories included lectins, putative antimicrobials, nematode allergens and stress-response genes. Consistent with previous studies describing a role in pathogen recognition for C-type lectins in *C. elegans* (Miltsch et al. 2014; O’Rourke et al. 2006), expression of lectins was high in bacterial treatments, as indicated by the up-regulation of two C-type lectin domain-containing proteins in 22-h *E. c*. (1.4 and 1.5) and in 24-h *B. a*. (0.6). Two concanavalin A-like lectins (lec-2 and lec-5) were up-regulated in 22-h *E. c*. (0.9 and 1.0) and 24-h *B. a*. (0.6), but down-regulated in 20-h *E. c*. (−0.7). C-type lectins may also be involved in regulating the interaction between the worm and its symbionts. In the marine nematode *Laxus oneistus*, the C-type lectin Mermaid is secreted on the surface of the worm where it mediates aggregation and attachment of bacterial ectosymbionts to the host’s surface (Bulgheresi et al. 2011).

Among putative antimicrobials, a transcript encoding Strictosidine synthase- a conserved enzyme involved in the biosynthesis of an alkaloid with antimicrobial activity (Sohani et al. 2009) was up-regulated in 22-h *E. c*. (1.1) and down-regulated in 36-h dsRNA (−0.7) while fatty acid synthase Fasn-1, that in *C. elegans* modulates antimicrobial peptide expression (Lee et al. 2010), was down-regulated in 22-h *E. c*. (−0.7).

Interestingly, treated worms exhibited increased expression of transcripts classified as allergens based on their ability to trigger an immune response in the human host (Kennedy 2000; Moreno et al. 2011), as well as stress-response genes and xenobiotic detoxicants. See ESM 3 for additional details.

### Summary

Overall, the data presented in this study show that the filarial nematode *B. malayi* is able to respond to experimental immune challenges, as revealed by the changes in the expression of several genes that in the free-living nematode *C. elegans* are involved in immunity. Similar to *C. elegans*, *B. malayi* possesses an innate immune system that lacks specialized immune cells such as macrophages and phagocytic cells, as well as canonical cytokine and chemokine signaling pathways (i.e., no NF-kB homolog has been found in *C. elegans*). The three main mechanisms of response to pathogens in *C. elegans* include avoidance guided by olfactory neurons that “sense” harmful bacteria and fungi (Pradel et al. 2007), mechanical barriers (i.e., cuticle and pharyngeal grinder), and production of inducible defenses such as antimicrobial peptides and reactive oxygen species. It is not clear yet whether the identification of pathogens is mediated by the activity of pathogen recognition receptors (PRRs) like TLR receptors, NOD receptors, and C-type lectins, that are able to recognize and bind to conserved motifs on the surface of pathogens (Akira et al. 2006). To date, only one TLR-like protein has been identified in *C. elegans* but it does not seem to be directly involved in pathogen recognition. One hypothesis is that surveillance mechanisms act by detecting disruption of critical core processes, such as translational inhibition, generated by bacterial infection (Dunbar et al. 2012).

Another open question is where the immune response takes place in *B. malayi*. In *C. elegans*, immunity signaling relies on epithelial cells in the intestine and in the epidermis. Epidermal immunity is induced by pathogen infection or mechanical damage and stimulates the production of antimicrobials via the p38 MAPK and the TGF-β pathway. In the intestine, in addition to the p38 MAPK pathway, the insulin signaling pathway mediates stress response and resistance against ingested pathogens. Due to its parasitic lifestyle, *B. malayi* is likely to be exposed to fewer pathogens compared to free living nematodes, as reflected by the lower diversity of its immune regulators (Murfin et al. 2012). In addition, the need to defend itself from the attack of the human host’s immune system may have shaped Brugia’s immune mechanisms differently from those of non-parasitic species. For example, the presence of antioxidant enzymes such as glutathione peroxidase in the cuticle has been seen as a strategy to avoid oxidative damage caused by leukocyte-derived reactive oxygen species (Cookson et al. 1992). Interestingly, a recent tissue-specific proteomic analysis revealed high abundance of immune-related proteins such as xenobiotic detoxicants in the intestine of adult *B. malayi* (Morris et al. 2015), suggesting that the intestine may be as critical as the cuticle in protecting the worm from pathogens and from the attack of human immune cells.

In summary, out of all the treatments included in this study, a 22-h exposure to *E. coli* lysate triggered the strongest response in *B. malayi*, displaying the highest number of DE transcripts compared to controls but also the strongest immune signature. A similar pattern, although less prominent, was found in worms exposed to *B. amyloliquefaciens* lysates. Surprisingly, exposure to 2.5 μM dsDNA appeared to be highly toxic to live adult female worms, as indicated by the sharp decline in motility after only 12 and 16 h of treatment. It is not clear why dsDNA had such a dramatic effect, as the DNA was obtained from a pure stock and sheared in RNAse-free water. Further studies testing the effects of different dsDNA concentrations need to be conducted to elucidate the mechanisms of apparent DNA toxicity in *B. malayi*. Further research is also needed in order to distinguish those expression changes exhibited by immune challenged worms that are a true antibacterial response from those that might represent a stress response against high xenobiotic concentrations in the medium.

Future studies will need to focus on the role of *Wolbachia* during the worm’s immune response. Current evidence suggests that *Wolbachia* acts as powerful modulator of human immunity, as indicated by its ability to delay apoptosis in human polymorphonuclear cells (PMNs) (Bazzocchi et al. 2007). In mosquitos, artificial infection with *Wolbachia* confers protection against pathogens (Ye et al. 2013) and it is possible that *Wolbachia* confers increased resistance to stress and pathogens to filarial nematodes as well. However, the same study found no evidence of similar protection in naturally-infected *Drosphila* populations. Similarly, previous studies did not detect any activation or suppression of inducible defenses in insects following *Wolbachia* infection (Bourtzis et al. 2000), indicating that this phenomenon is highly dependent on the nature of the symbiosis. As *Wolbachia* resides in cytoplasmic host-derived vacuoles, one possibility is that it avoids phagocytic lysis and degradation either by modifying the vacuoles and/or releasing effector molecules to manipulate the phagocytic pathway. Interestingly, a similar mechanism has been described for *Coxiella burnetii*, an intracellular bacterium that also develops in phagolysosomal-like vacuoles. *C. burnetii* interacts with both the phagocytic and autophagic pathways to avoid fusion of the phagosomal vacuole with lysosomes, and uses autophagic vesicles to obtain key nutrients required for its development (Romano et al. 2007). In order to determine whether similar mechanisms underlie the relationship between the autophagy pathway and *Wolbachia* populations in filarial nematodes, future research is needed to reveal currently unknown aspects of the symbiosis, including the regulatory networks that control *Brugia*’s innate immune pathways.

## Conclusions

Transcriptome profiles of *B. malayi* after immune challenge reveal that the filarial nematode, similarly to *C. elegans*, possesses an array of inducible defenses that are activated upon stress and/or pathogen exposure. Examples of these defenses are conserved signaling pathways including those of p38 MAPK, TGF-β and insulin and small RNAs. It is likely that these pathways act in concert to modulate the tradeoffs between immune activation and cell homeostasis. Even with its challenging technical limitations, this study provides a starting point to address a largely overlooked field, the immunity of filarial nematodes, and offers the potential to improve our understanding of the symbiosis between *B. malayi* and *Wolbachia*

## Electronic supplementary material

Below is the link to the electronic supplementary material.

ESM 1 (PDF 78 kb)

ESM 2 (PDF 197 kb)

ESM 3 (PDF 216 kb)
