# Supplementary material for: Characterization of innate immunity genes in the parasitic nematode Brugia malayi
Source: Symbiosis. 2016 Jan 5;68:145–55. doi: 10.1007/s13199-015-0374-7 (PMC4826884; doi:10.1007/s13199-015-0374-7)
Supplement: Supplementary file 1 — (PDF 78 kb) [file 13199_2015_374_MOESM1_ESM.pdf]

Electronic Supplementary Material 1

Journal: Symbiosis

Characterization of innate immune genes in the parasitic nematode *Brugia malayi*

Silvia Libro[1], Barton E. Slatko, Jeremy M. Foster

New England Biolabs, Inc., Genome Biology Division, 240 County Road, Ipswich, MA USA 01938

[1] Corresponding author: libro@neb.com - Ph: 978-380-7311

Table S1. Summary of transcriptome statistics for the 22 cDNA libraries of *B. malayi* adult females used in this study. *: only one replicate was included in this study.

Treatment	Raw read pairs	Retained reads	Mapped read pairs	% Concordant pairs
24-h C+A_1	11,522,190	8,191,312	7,299,994	88.0%
24-h C+A_2	11,149,279	7,979,214	7,141,607	88.2%
36-h C+A_1	9,893,992	7,115,651	6,332,755	87.6%
36-h C+A_2	27,215,372	26,894,403	22,170,729	27.80%
24-h C-A_1	9,816,930	7,086,969	6,351,339	88.2%
24-h C-A_2	27,827,879	23,376,037	21,250,035	89.8%
36-h C-A*	32,318,900	32,057,828	27,877,923	83.90%
12-h DNA_1	17,752,956	12,360,679	11,043,591	88.50%
12-h DNA_2	14,819,022	14,076,695	12,188,303	85.80%
16-h DNA*	26,966,257	22,265,287	19,343,877	86.10%
24-h dsRNA_1	12,317,919	8,936,723	7,694,675	84.50%
24-h dsRNA_2	5,525,928	3,517,665	2,999,998	84.00%
36-h dsRNA_1	18,227,322	13,055,938	11,612,336	87.30%
36-h dsRNA_2	43,543,117	37,338,685	34,195,466	89.50%
20-h *E.c.*_1	12,959,314	9,125,431	6,731,359	73.30%
20-h *E.c.*_2	22,122,249	15,348,791	13,245,423	85.2%
22-h *E.c.*_1	5,744,838	3,828,544	3,256,067	83.7%
22-h *E.c.*_2	26,338,100	22,023,388	19,229,078	86.5%
24-h *B.a.*_1	9,789,699	6,993,493	6,217,349	87.6
24-h *B.a.*_2	10,915,760	7,791,417	7,004,993	88.6
36-h *B.a.*_1	38,999,794	31,305,052	25,293,670	78.6
36-h *B.a.*_2	27,101,591	23,011,535	18,001,872	77.1
