# Supplementary material for: Characterization of innate immunity genes in the parasitic nematode Brugia malayi
Source: Symbiosis. 2016 Jan 5;68:145–55. doi: 10.1007/s13199-015-0374-7 (PMC4826884; doi:10.1007/s13199-015-0374-7)
Supplement: Supplementary file 2 — (PDF 197 kb) [file 13199_2015_374_MOESM2_ESM.pdf]

Electronic Supplementary Material 2
Journal: Symbiosis
Characterization of innate immune genes in the parasitic nematode *Brugia malayi*
Silvia Libro, Barton E. Slatko, Jeremy M. Foster
New England Biolabs, Inc., Genome Biology Division, 240 County Road, Ipswich, MA USA 01938
Corresponding author: libro@neb.com - Ph: 978-380-7311

Table S2. Functional annotation clustering and enrichment analysis of DE transcripts.

	Category	Term	Count	%	PValue	Genes	List Total	Pop Hits	Pop Total	Fold Enrichment	Bonferroni	Benjamini	FDR
						**12-h dsDNA**							
Annotation Cluster 1	GOTERM_BP_FAT	GO:0007155~cell adhesion	4	5.71	0.01	2294482, 2296386, 2298009, 2290446	17	6	198	7.76	0.37	0.37	7.33
Enrichment Score: 1.84	GOTERM_BP_FAT	GO:0022610~biological adhesion	4	5.71	0.01	2294482, 2296386, 2298009, 2290446	17	6	198	7.76	0.37	0.37	7.33
	GOTERM_BP_FAT	GO:0007160~cell-matrix adhesion	3	4.29	0.02	2294482, 2296386, 2290446	17	3	198	11.65	0.66	0.42	16.34
	GOTERM_BP_FAT	GO:0031589~cell-substrate adhesion	3	4.29	0.02	2294482, 2296386, 2290446	17	3	198	11.65	0.66	0.42	16.34
	INTERPRO	IPR013032:EGF-like region, conserved site	6	8.57	0.04	2294482, 2296386, 2291857, 2300247, 2290446, 2294634	57	18	512	2.99	0.99	0.90	35.01
Annotation Cluster 2	INTERPRO	IPR000742:EGF-like, type 3	6	8.57	0.01	2296386, 2291857, 2300247, 2294112, 2290446, 2294634	57	14	512	3.85	0.77	0.77	13.10
Enrichment Score: 1.28	INTERPRO	IPR013032:EGF-like region, conserved site	6	8.57	0.04	2294482, 2296386, 2291857, 2300247, 2290446, 2294634	57	18	512	2.99	0.99	0.90	35.01
	SP_PIR_KEYWORDS	egf-like domain	5	7.14	0.05	2296386, 2291857, 2300247, 2294112, 2290446	31	12	240	3.23	0.84	0.84	35.83
	INTERPRO	IPR006210:EGF-like	5	7.14	0.06	2296386, 2291857, 2300247, 2294112, 2294634	57	14	512	3.21	1.00	0.82	48.33
	INTERPRO	IPR000152:EGF-type aspartate/asparagine hydroxylation conserved site	4	5.71	0.06	2296386, 2291857, 2294112, 2290446	57	9	512	3.99	1.00	0.80	53.61
	SMART	SM00181:EGF	5	7.14	0.10	2296386, 2291857, 2300247, 2294112, 2294634	34	14	246	2.58	0.98	0.86	61.61
	INTERPRO	IPR006209:EGF	3	4.29	0.13	2291857, 2300247, 2294112	57	6	512	4.49	1.00	0.94	80.42
Annotation Cluster 3	PIR_SUPERFAMILY	PIRSF038285:cuticle collagen	3	4.29	0.07	2298208, 2292335, 2291120	8	5	70	5.25	0.37	0.37	31.05
Enrichment Score: 0.76	INTERPRO	IPR008160:Collagen triple helix repeat	3	4.29	0.13	2298208, 2292335, 2291120	57	6	512	4.49	1.00	0.94	80.42
	GOTERM_MF_FAT	GO:0042302~structural constituent of cuticle	3	4.29	0.17	2298208, 2292335, 2291120	31	7	279	3.86	1.00	1.00	85.86
	INTERPRO	IPR002486:Nematode cuticle collagen, N-terminal	3	4.29	0.17	2298208, 2292335, 2291120	57	7	512	3.85	1.00	0.96	88.66
	SP_PIR_KEYWORDS	collagen	3	4.29	0.21	2298208, 2292335, 2291120	31	7	240	3.32	1.00	0.99	88.38
	GOTERM_MF_FAT	GO:0005198~structural molecule activity	3	4.29	0.46	2298208, 2292335, 2291120	31	14	279	1.93	1.00	1.00	99.86
Annotation Cluster 4	GOTERM_MF_FAT	GO:0003774~motor activity	3	4.29	0.09	2294983, 2292010, 2296937	31	5	279	5.40	1.00	1.00	64.05
Enrichment Score: 0.69	GOTERM_CC_FAT	GO:0044430~cytoskeletal part	3	4.29	0.13	2294983, 2292010, 2296937	12	6	99	4.13	0.96	0.96	67.37
	GOTERM_CC_FAT	GO:0043228~non-membrane-bounded organelle	4	5.71	0.28	2292671, 2294983, 2292010, 2296937	12	17	99	1.94	1.00	0.98	92.94
	GOTERM_CC_FAT	GO:0043232~intracellular non-membrane-bounded organelle	4	5.71	0.28	2292671, 2294983, 2292010, 2296937	12	17	99	1.94	1.00	0.98	92.94
	GOTERM_CC_FAT	GO:0005856~cytoskeleton	3	4.29	0.35	2294983, 2292010, 2296937	12	11	99	2.25	1.00	0.96	96.80
Annotation Cluster 5	GOTERM_MF_FAT	GO:0005509~calcium ion binding	4	5.71	0.18	2293851, 2291857, 2298009, 2290446	31	14	279	2.57	1.00	1.00	88.18
Enrichment Score: 0.35	GOTERM_MF_FAT	GO:0046872~metal ion binding	9	12.86	0.43	2300013, 2293547, 2293851, 2291857, 2294308, 2298009, 2290446, 2296172, 2298196	31	67	279	1.21	1.00	1.00	99.78
	GOTERM_MF_FAT	GO:0043167~ion binding	9	12.86	0.51	2300013, 2293547, 2293851, 2291857, 2294308, 2298009, 2290446, 2296172, 2298196	31	71	279	1.14	1.00	1.00	99.95
	GOTERM_MF_FAT	GO:0043169~cation binding	9	12.86	0.51	2300013, 2293547, 2293851, 2291857, 2294308, 2298009, 2290446, 2296172, 2298196	31	71	279	1.14	1.00	1.00	99.95
	GOTERM_MF_FAT	GO:0046914~transition metal ion binding	5	7.14	0.84	2300013, 2293547, 2294308, 2296172, 2298196	31	51	279	0.88	1.00	1.00	100.00
Annotation Cluster 6	GOTERM_MF_FAT	GO:0005524~ATP binding	3	4.29	0.93	2294308, 2292010, 2296937	31	36	279	0.75	1.00	1.00	100.00
Enrichment Score: 0.02	GOTERM_MF_FAT	GO:0032559~adenyl ribonucleotide binding	3	4.29	0.93	2294308, 2292010, 2296937	31	36	279	0.75	1.00	1.00	100.00
	GOTERM_MF_FAT	GO:0030554~adenyl nucleotide binding	3	4.29	0.93	2294308, 2292010, 2296937	31	37	279	0.73	1.00	1.00	100.00
	GOTERM_MF_FAT	GO:0001882~nucleoside binding	3	4.29	0.93	2294308, 2292010, 2296937	31	37	279	0.73	1.00	1.00	100.00
	GOTERM_MF_FAT	GO:0001883~purine nucleoside binding	3	4.29	0.93	2294308, 2292010, 2296937	31	37	279	0.73	1.00	1.00	100.00
	GOTERM_MF_FAT	GO:0032553~ribonucleotide binding	3	4.29	0.96	2294308, 2292010, 2296937	31	41	279	0.66	1.00	1.00	100.00
	GOTERM_MF_FAT	GO:0032555~purine ribonucleotide binding	3	4.29	0.96	2294308, 2292010, 2296937	31	41	279	0.66	1.00	1.00	100.00
	GOTERM_MF_FAT	GO:0017076~purine nucleotide binding	3	4.29	0.96	2294308, 2292010, 2296937	31	42	279	0.64	1.00	1.00	100.00
	GOTERM_MF_FAT	GO:0000166~nucleotide binding	3	4.29	0.99	2294308, 2292010, 2296937	31	51	279	0.53	1.00	1.00	100.00
						**16-h dsDNA**							
Annotation Cluster 1	SP_PIR_KEYWORDS	dna-binding	3	7.14	0.27	2298392, 2292671, 2298196	15	230	3152	2.74	1.00	1.00	92.62
Enrichment Score: 0.44	SP_PIR_KEYWORDS	nucleus	3	7.14	0.28	2298392, 2292671, 2298196	15	232	3152	2.72	1.00	0.98	92.90
	GOTERM_MF_FAT	GO:0003677~DNA binding	3	7.14	0.63	2298392, 2292671, 2298196	17	519	4047	1.38	1.00	1.00	100.00
Annotation Cluster 2	GOTERM_MF_FAT	GO:0046914~transition metal ion binding	4	9.52	0.67	2300013, 2299149, 2296172, 2298196	17	833	4047	1.14	1.00	1.00	100.00
Enrichment Score: 0.11	GOTERM_MF_FAT	GO:0046872~metal ion binding	4	9.52	0.82	2300013, 2299149, 2296172, 2298196	17	1032	4047	0.92	1.00	1.00	100.00
	GOTERM_MF_FAT	GO:0043167~ion binding	4	9.52	0.83	2300013, 2299149, 2296172, 2298196	17	1050	4047	0.91	1.00	1.00	100.00
	GOTERM_MF_FAT	GO:0043169~cation binding	4	9.52	0.83	2300013, 2299149, 2296172, 2298196	17	1050	4047	0.91	1.00	1.00	100.00
						**24-h dsRNA**							
Annotation Cluster 1	INTERPRO	IPR013032:EGF-like region, conserved site	6	8.57	0.00	2294482, 2296386, 2291857, 2300247, 2290446, 2294634	57	92	7207	8.25	0.08	0.04	0.80
Enrichment Score: 2.99	GOTERM_BP_FAT	GO:0007160~cell-matrix adhesion	3	4.29	0.00	2294482, 2296386, 2290446	17	8	2832	62.47	0.05	0.05	0.82
	GOTERM_BP_FAT	GO:0031589~cell-substrate adhesion	3	4.29	0.00	2294482, 2296386, 2290446	17	8	2832	62.47	0.05	0.05	0.82
	GOTERM_BP_FAT	GO:0007155~cell adhesion	4	5.71	0.00	2294482, 2296386, 2298009, 2290446	17	43	2832	15.50	0.09	0.05	1.59
	GOTERM_BP_FAT	GO:0022610~biological adhesion	4	5.71	0.00	2294482, 2296386, 2298009, 2290446	17	43	2832	15.50	0.09	0.05	1.59
Annotation Cluster 2	INTERPRO	IPR000742:EGF-like, type 3	6	8.57	0.00	2296386, 2291857, 2300247, 2294112, 2290446, 2294634	57	56	7207	13.55	0.01	0.01	0.08
Enrichment Score: 2.79	INTERPRO	IPR013032:EGF-like region, conserved site	6	8.57	0.00	2294482, 2296386, 2291857, 2300247, 2290446, 2294634	57	92	7207	8.25	0.08	0.04	0.80

	SP_PIR_KEYWORDS	egf-like domain	5	7.14	0.00	2296386, 2291857, 2300247, 2294112, 2290446	31	57	3152	8.92	0.07	0.07	1.65
	INTERPRO	IPR006210:EGF-like	5	7.14	0.00	2296386, 2291857, 2300247, 2294112, 2294634	57	69	7207	9.16	0.21	0.08	2.21
	INTERPRO	IPR000152:EGF-type aspartate/asparagine hydroxylation conserved site	4	5.71	0.00	2296386, 2291857, 2294112, 2290446	57	34	7207	14.88	0.24	0.07	2.55
	SMART	SM00181:EGF	5	7.14	0.00	2296386, 2291857, 2300247, 2294112, 2294634	34	69	3277	6.98	0.16	0.16	4.10
	INTERPRO	IPR006209:EGF	3	4.29	0.02	2291857, 2300247, 2294112	57	28	7207	13.55	0.91	0.29	20.44
Annotation Cluster 3	PIR_SUPERFAMILY	PIRSF038285:cuticle collagen	3	4.29	0.01	2298208, 2292335, 2291120	8	24	1091	17.05	0.05	0.05	4.29
Enrichment Score: 1.10	GOTERM_MF_FAT	GO:0042302~structural constituent of cuticle	3	4.29	0.04	2298208, 2292335, 2291120	31	44	4047	8.90	0.97	0.97	36.45
	INTERPRO	IPR002486:Nematode cuticle collagen, N-terminal	3	4.29	0.05	2298208, 2292335, 2291120	57	44	7207	8.62	1.00	0.51	41.50
	INTERPRO	IPR008160:Collagen triple helix repeat	3	4.29	0.15	2298208, 2292335, 2291120	57	88	7207	4.31	1.00	0.89	84.37
	SP_PIR_KEYWORDS	collagen	3	4.29	0.18	2298208, 2292335, 2291120	31	82	3152	3.72	1.00	0.92	83.66
	GOTERM_MF_FAT	GO:0005198~structural molecule activity	3	4.29	0.55	2298208, 2292335, 2291120	31	244	4047	1.61	1.00	1.00	99.98
Annotation Cluster 4	GOTERM_MF_FAT	GO:0003774~motor activity	3	4.29	0.04	2294983, 2292010, 2296937	31	44	4047	8.90	0.97	0.97	36.45
Enrichment Score: 0.65	GOTERM_CC_FAT	GO:0044430~cytoskeletal part	3	4.29	0.12	2294983, 2292010, 2296937	12	74	1317	4.45	0.95	0.95	64.79
	GOTERM_CC_FAT	GO:0005856~cytoskeleton	3	4.29	0.31	2294983, 2292010, 2296937	12	135	1317	2.44	1.00	0.99	94.92
	GOTERM_CC_FAT	GO:0043228~non-membrane-bounded organelle	4	5.71	0.59	2292671, 2294983, 2292010, 2296937	12	347	1317	1.27	1.00	0.98	99.91
	GOTERM_CC_FAT	GO:0043232~intracellular non-membrane-bounded organelle	4	5.71	0.59	2292671, 2294983, 2292010, 2296937	12	347	1317	1.27	1.00	0.98	99.91
Annotation Cluster 5	GOTERM_MF_FAT	GO:0005509~calcium ion binding	4	5.71	0.08	2293851, 2291857, 2298009, 2290446	31	137	4047	3.81	1.00	0.97	58.71
Enrichment Score: 0.40	GOTERM_MF_FAT	GO:0046872~metal ion binding	9	12.86	0.51	2300013, 2293547, 2293851, 2291857, 2294308, 2298009, 2290446, 2296172, 2298196	31	1032	4047	1.14	1.00	1.00	99.95
	GOTERM_MF_FAT	GO:0043167~ion binding	9	12.86	0.53	2300013, 2293547, 2293851, 2291857, 2294308, 2298009, 2290446, 2296172, 2298196	31	1050	4047	1.12	1.00	1.00	99.97
	GOTERM_MF_FAT	GO:0043169~cation binding	9	12.86	0.53	2300013, 2293547, 2293851, 2291857, 2294308, 2298009, 2290446, 2296172, 2298196	31	1050	4047	1.12	1.00	1.00	99.97
	GOTERM_MF_FAT	GO:0046914~transition metal ion binding	5	7.14	0.89	2300013, 2293547, 2294308, 2296172, 2298196	31	833	4047	0.78	1.00	1.00	100.00
Annotation Cluster 6	GOTERM_MF_FAT	GO:0005524~ATP binding	3	4.29	0.96	2294308, 2292010, 2296937	31	629	4047	0.62	1.00	1.00	100.00
Enrichment Score: 0.01	GOTERM_MF_FAT	GO:0032559~adenyl ribonucleotide binding	3	4.29	0.96	2294308, 2292010, 2296937	31	630	4047	0.62	1.00	1.00	100.00
	GOTERM_MF_FAT	GO:0030554~adenyl nucleotide binding	3	4.29	0.97	2294308, 2292010, 2296937	31	660	4047	0.59	1.00	1.00	100.00
	GOTERM_MF_FAT	GO:0001883~purine nucleoside binding	3	4.29	0.97	2294308, 2292010, 2296937	31	660	4047	0.59	1.00	1.00	100.00
	GOTERM_MF_FAT	GO:0001882~nucleoside binding	3	4.29	0.97	2294308, 2292010, 2296937	31	664	4047	0.59	1.00	1.00	100.00
	GOTERM_MF_FAT	GO:0032553~ribonucleotide binding	3	4.29	0.99	2294308, 2292010, 2296937	31	770	4047	0.51	1.00	1.00	100.00
	GOTERM_MF_FAT	GO:0032555~purine ribonucleotide binding	3	4.29	0.99	2294308, 2292010, 2296937	31	770	4047	0.51	1.00	1.00	100.00
	GOTERM_MF_FAT	GO:0017076~purine nucleotide binding	3	4.29	0.99	2294308, 2292010, 2296937	31	806	4047	0.49	1.00	1.00	100.00
	GOTERM_MF_FAT	GO:0000166~nucleotide binding	3	4.29	1.00	2294308, 2292010, 2296937	31	963	4047	0.41	1.00	1.00	100.00

36-h dsRNA

Annotation Cluster 1	GOTERM_MF_FAT	GO:0046872~metal ion binding	6	26.09	0.04	2292733, 2297343, 2296172, 2294057, 2293642, 2296390	10	67	279	2.50	0.67	0.67	28.33
Enrichment Score: 0.96	GOTERM_MF_FAT	GO:0043167~ion binding	6	26.09	0.05	2292733, 2297343, 2296172, 2294057, 2293642, 2296390	10	71	279	2.36	0.76	0.51	34.66
	GOTERM_MF_FAT	GO:0043169~cation binding	6	26.09	0.05	2292733, 2297343, 2296172, 2294057, 2293642, 2296390	10	71	279	2.36	0.76	0.51	34.66
	GOTERM_MF_FAT	GO:0046914~transition metal ion binding	5	21.74	0.06	2297343, 2296172, 2294057, 2293642, 2296390	10	51	279	2.74	0.83	0.45	41.19
	SP_PIR_KEYWORDS	zinc	3	13.04	0.24	2297343, 2294057, 2293642	9	28	240	2.86	0.99	0.99	86.31
	SP_PIR_KEYWORDS	metal-binding	3	13.04	0.30	2297343, 2294057, 2293642	9	33	240	2.42	1.00	0.96	93.00
	GOTERM_MF_FAT	GO:0008270~zinc ion binding	3	13.04	0.42	2297343, 2294057, 2293642	10	43	279	1.95	1.00	0.98	98.89

20-h *E.c.*

Annotation Cluster 1	INTERPRO	IPR002486:Nematode cuticle collagen, N-terminal	3	11.11	0.01	A8PUG1, A8P9P7, A8NV36	23	44	7207	21.36	0.34	0.34	7.34
Enrichment Score: 1.63	GOTERM_MF_FAT	GO:0042302~structural constituent of cuticle	3	11.11	0.01	A8PUG1, A8P9P7, A8NV36	14	44	4047	19.71	0.27	0.27	7.30
	SP_PIR_KEYWORDS	collagen	3	11.11	0.02	A8PUG1, A8P9P7, A8NV36	10	82	3152	11.53	0.28	0.28	13.95
	INTERPRO	IPR008160:Collagen triple helix repeat	3	11.11	0.03	A8PUG1, A8P9P7, A8NV36	23	88	7207	10.68	0.79	0.54	25.01
	GOTERM_MF_FAT	GO:0005198~structural molecule activity	3	11.11	0.18	A8PUG1, A8P9P7, A8NV36	14	244	4047	3.55	1.00	0.98	83.84

22-h *E.c.*

Annotation Cluster 1	INTERPRO	IPR000742:EGF-like, type 3	12	4.03	0.00	2289402, 2293903, 2296386, 2291857, 2292811, 2294910, 2300247, 2294112, 2294454, 2290446, 2294634, 2298638	251	56	7207	6.15	0.00	0.00	0.00
Enrichment Score: 3.64	INTERPRO	IPR006210:EGF-like	11	3.69	0.00	2289402, 2293903, 2296386, 2291857, 2292811, 2294910, 2300247, 2294112, 2294454, 2294634, 2298638	251	69	7207	4.58	0.05	0.02	0.16
	SP_PIR_KEYWORDS	egf-like domain	10	3.36	0.00	2289402, 2296386, 2291857, 2292811, 2294910, 2300247, 2294112, 2294454, 2290446, 2298638	113	57	3152	4.89	0.01	0.01	0.15
	SMART	SM00181:EGF	11	3.69	0.00	2289402, 2293903, 2296386, 2291857, 2292811, 2294910, 2300247, 2294112, 2294454, 2294634, 2298638	121	69	3277	4.32	0.02	0.02	0.18
	INTERPRO	IPR013032:EGF-like region, conserved site	12	4.03	0.00	2293903, 2294482, 2296386, 2291857, 2292811, 2294910, 2300247, 2294454, 2290446, 2300095, 2294634, 2298638	251	92	7207	3.75	0.12	0.04	0.44
	INTERPRO	IPR000152:EGF-type aspartate/asparagine hydroxylation conserved site	7	2.35	0.00	2289402, 2296386, 2291857, 2294112, 2294454, 2290446, 2298638	251	34	7207	5.91	0.33	0.08	1.35
	INTERPRO	IPR006209:EGF	5	1.68	0.02	2293903, 2291857, 2300247, 2294112, 2294454	251	28	7207	5.13	1.00	0.43	19.07
Annotation Cluster 2	GOTERM_CC_FAT	GO:0016021~integral to membrane	29	9.73	0.00	2293786, 2300676, 2297259, 2290374, 2294482, 2291727, 2291279, 2295322, 2300636, 2289525, 2298638, 2296588, 2291958, 2294159, 2297956, 2295347, 2294340, 2293300, 2292515, 2289491, 2297692, 2292683, 2295396, 2293165, 2296455, 2291073, 2296494, 2297177, 2299912	45	519	1317	1.64	0.04	0.02	0.77
Enrichment Score: 2.55	GOTERM_CC_FAT	GO:0031224~intrinsic to membrane	29	9.73	0.00	2293786, 2300676, 2297259, 2290374, 2294482, 2291727, 2291279, 2295322, 2300636, 2289525, 2298638, 2296588, 2291958, 2294159, 2297956, 2295347, 2294340, 2293300, 2292515, 2289491, 2297692, 2292683, 2295396, 2293165, 2296455, 2291073, 2296494, 2297177, 2299912	45	527	1317	1.61	0.05	0.02	1.03
	SP_PIR_KEYWORDS	transmembrane	19	6.38	0.02	2293786, 2297259, 2290374, 2295322, 2298009, 2292515, 2289525, 2297692, 2292683, 2298638, 2296588, 2291958, 2293165, 2296455, 2297956, 2291073, 2295347, 2299912, 2294340	113	311	3152	1.70	0.87	0.40	22.92

Annotation Cluster 3	GOTERM_MF_FAT	GO:0070011~peptidase activity, acting on L-amino acid peptides	17	5.70	0.00	2299679, 2293573, 2299491, 2300095, 2296172, 2294534, 2290176, 2291371, 2292564, 2293547, 2292565, 2295209, 2297765, 2297330, 2289611, 2292649, 2293668	137	188	4047	2.67	0.09	0.09	0.59
Enrichment Score: 2.54	GOTERM_MF_FAT	GO:0004175~endopeptidase activity	12	4.03	0.00	2299679, 2292564, 2293573, 2292565, 2295209, 2297765, 2297330, 2289611, 2292649, 2300095, 2293668, 2291371	137	122	4047	2.91	0.39	0.22	2.96
	GOTERM_MF_FAT	GO:0008233~peptidase activity	17	5.70	0.00	2299679, 2293573, 2299491, 2300095, 2296172, 2294534, 2290176, 2291371, 2292564, 2293547, 2292565, 2295209, 2297765, 2297330, 2289611, 2292649, 2293668	137	219	4047	2.29	0.40	0.16	3.03
	GOTERM_MF_FAT	GO:0008237~metallopeptidase activity	9	3.02	0.00	2293547, 2293573, 2292565, 2295209, 2297765, 2297330, 2299491, 2300095, 2293668	137	75	4047	3.54	0.50	0.16	4.14
	GOTERM_BP_FAT	GO:0006508~proteolysis	17	5.70	0.01	2299679, 2293573, 2299491, 2300095, 2294534, 2290176, 2291371, 2292564, 2293547, 2292565, 2295209, 2297765, 2297330, 2289611, 2292649, 2293668, 2294063	94	245	2832	2.09	0.61	0.61	6.22
	SP_PIR_KEYWORDS	Protease	11	3.69	0.01	2299679, 2292564, 2292565, 2295209, 2297765, 2297330, 2289611, 2292649, 2300095, 2290176, 2291371	113	112	3152	2.74	0.38	0.21	5.99
	GOTERM_MF_FAT	GO:0004222~metalloendopeptidase activity	7	2.35	0.01	2293573, 2292565, 2295209, 2297765, 2297330, 2300095, 2293668	137	50	4047	4.14	0.72	0.22	7.39
Annotation Cluster 4	GOTERM_BP_FAT	GO:0022610~biological adhesion	6	2.01	0.01	2299418, 2294482, 2296386, 2298009, 2290446, 2298638	94	43	2832	4.20	0.89	0.67	14.12
Enrichment Score: 1.75	GOTERM_BP_FAT	GO:0007155~cell adhesion	6	2.01	0.01	2299418, 2294482, 2296386, 2298009, 2290446, 2298638	94	43	2832	4.20	0.89	0.67	14.12
	GOTERM_BP_FAT	GO:0007160~cell-matrix adhesion	3	1.01	0.03	2294482, 2296386, 2290446	94	8	2832	11.30	0.99	0.79	27.90
	GOTERM_BP_FAT	GO:0031589~cell-substrate adhesion	3	1.01	0.03	2294482, 2296386, 2290446	94	8	2832	11.30	0.99	0.79	27.90
Annotation Cluster 5	INTERPRO	IPR013151:Immunoglobulin	6	2.01	0.00	2296981, 2294830, 2296377, 2295910, 2300340, 2300096	251	24	7207	7.18	0.38	0.08	1.66
Enrichment Score: 1.58	INTERPRO	IPR007110:Immunoglobulin-like	8	2.68	0.01	2296981, 2294830, 2293920, 2296377, 2295910, 2297728, 2300340, 2300096	251	66	7207	3.48	0.95	0.32	10.06
	INTERPRO	IPR013783:Immunoglobulin-like fold	8	2.68	0.01	2296981, 2294830, 2293920, 2296377, 2295910, 2297728, 2300340, 2300096	251	67	7207	3.43	0.96	0.31	10.87
	INTERPRO	IPR003598:Immunoglobulin subtype 2	6	2.01	0.02	2296981, 2294830, 2296377, 2295910, 2297728, 2300096	251	47	7207	3.67	1.00	0.48	27.34
	SMART	SM00408:IGc2	6	2.01	0.03	2296981, 2294830, 2296377, 2295910, 2297728, 2300096	121	47	3277	3.46	0.93	0.49	26.32
	INTERPRO	IPR003599:Immunoglobulin subtype	4	1.34	0.16	2296981, 2296377, 2295910, 2300340	251	40	7207	2.87	1.00	0.93	91.32
	INTERPRO	IPR013098:Immunoglobulin I-set	4	1.34	0.18	2293920, 2296377, 2295910, 2297728	251	42	7207	2.73	1.00	0.93	93.50
	SMART	SM00409:IG	4	1.34	0.18	2296981, 2296377, 2295910, 2300340	121	40	3277	2.71	1.00	0.94	88.74
Annotation Cluster 6	INTERPRO	IPR000731:Sterol-sensing 5TM box	4	1.34	0.05	2295396, 2300676, 2294159, 2289491	251	24	7207	4.79	1.00	0.64	50.30
Enrichment Score: 1.27	GOTERM_MF_FAT	GO:0008158~hedgehog receptor activity	4	1.34	0.05	2295396, 2300676, 2294159, 2289491	137	26	4047	4.54	1.00	0.69	50.96
	INTERPRO	IPR003392:Patched	4	1.34	0.06	2295396, 2300676, 2294159, 2289491	251	26	7207	4.42	1.00	0.70	57.64
Annotation Cluster 7	GOTERM_MF_FAT	GO:0004222~metalloendopeptidase activity	7	2.35	0.01	2293573, 2292565, 2295209, 2297765, 2297330, 2300095, 2293668	137	50	4047	4.14	0.72	0.22	7.39
Enrichment Score: 1.16	INTERPRO	IPR006026:Peptidase, metallopeptidases	3	1.01	0.12	2295209, 2297330, 2300095	251	17	7207	5.07	1.00	0.88	82.13
	SMART	SM00235:ZnMc	3	1.01	0.13	2295209, 2297330, 2300095	121	17	3277	4.78	1.00	0.89	77.67
	SP_PIR_KEYWORDS	metalloprotease	3	1.01	0.25	2295209, 2297330, 2300095	113	27	3152	3.10	1.00	0.79	95.33
Annotation Cluster 8	INTERPRO	IPR000152:EGF-type aspartate/asparagine hydroxylation conserved site	7	2.35	0.00	2289402, 2296386, 2291857, 2294112, 2294454, 2290446, 2298638	251	34	7207	5.91	0.33	0.08	1.35
Enrichment Score: 1.13	GOTERM_MF_FAT	GO:0005509~calcium ion binding	8	2.68	0.18	2293851, 2291857, 2298009, 2295347, 2294454, 2290446, 2296422, 2298638	137	137	4047	1.72	1.00	0.91	91.30
	INTERPRO	IPR018097:EGF-like calcium-binding, conserved site	3	1.01	0.21	2291857, 2294454, 2290446	251	25	7207	3.45	1.00	0.96	96.58
	INTERPRO	IPR001881:EGF-like calcium-binding	3	1.01	0.24	2291857, 2294454, 2290446	251	27	7207	3.19	1.00	0.97	97.86
	SMART	SM00179:EGF_CA	3	1.01	0.26	2291857, 2294454, 2290446	121	27	3277	3.01	1.00	0.96	96.44
Annotation Cluster 9	GOTERM_MF_FAT	GO:0030594~neurotransmitter receptor activity	5	1.68	0.02	2290374, 2296455, 2292515, 2289525, 2299912	137	29	4047	5.09	0.96	0.41	17.45
Enrichment Score: 1.08	GOTERM_MF_FAT	GO:0042165~neurotransmitter binding	5	1.68	0.02	2290374, 2296455, 2292515, 2289525, 2299912	137	29	4047	5.09	0.96	0.41	17.45
	SP_PIR_KEYWORDS	cell membrane	5	1.68	0.02	2296455, 2292515, 2289525, 2299912, 2298638	113	31	3152	4.50	0.85	0.47	21.78
	INTERPRO	IPR006202:Neurotransmitter-gated ion-channel ligand-binding	5	1.68	0.02	2293300, 2296455, 2292515, 2289525, 2299912	251	32	7207	4.49	1.00	0.48	28.49
	SP_PIR_KEYWORDS	transmembrane	19	6.38	0.02	2293786, 2297259, 2290374, 2295322, 2298009, 2292515, 2289525, 2297692, 2292683, 2298638, 2296588, 2291958, 2293165, 2296455, 2297956, 2291073, 2295347, 2299912, 2294340	113	311	3152	1.70	0.87	0.40	22.92
	GOTERM_CC_FAT	GO:0005886~plasma membrane	8	2.68	0.03	2294482, 2296455, 2292515, 2295347, 2289525, 2295407, 2299912, 2298638	45	91	1317	2.57	0.72	0.27	23.26
	INTERPRO	IPR018000:Neurotransmitter-gated ion-channel, conserved site	4	1.34	0.04	2296455, 2292515, 2289525, 2299912	251	22	7207	5.22	1.00	0.59	42.68
	INTERPRO	IPR006201:Neurotransmitter-gated ion-channel	5	1.68	0.04	2293300, 2296455, 2292515, 2289525, 2299912	251	38	7207	3.78	1.00	0.60	44.66
	GOTERM_MF_FAT	GO:0005230~extracellular ligand-gated ion channel activity	5	1.68	0.04	2293300, 2296455, 2292515, 2289525, 2299912	137	40	4047	3.69	1.00	0.74	43.16
	GOTERM_CC_FAT	GO:0045211~postsynaptic membrane	5	1.68	0.04	2293300, 2296455, 2292515, 2289525, 2299912	45	41	1317	3.57	0.87	0.34	34.89
	SP_PIR_KEYWORDS	postsynaptic cell membrane	4	1.34	0.05	2296455, 2292515, 2289525, 2299912	113	23	3152	4.85	0.98	0.54	39.69
	INTERPRO	IPR006029:Neurotransmitter-gated ion-channel transmembrane region	4	1.34	0.05	2296455, 2292515, 2289525, 2299912	251	24	7207	4.79	1.00	0.64	50.30
	GOTERM_MF_FAT	GO:0015276~ligand-gated ion channel activity	5	1.68	0.05	2293300, 2296455, 2292515, 2289525, 2299912	137	42	4047	3.52	1.00	0.74	48.31
	GOTERM_MF_FAT	GO:0022834~ligand-gated channel activity	5	1.68	0.05	2293300, 2296455, 2292515, 2289525, 2299912	137	42	4047	3.52	1.00	0.74	48.31
	SP_PIR_KEYWORDS	synapse	4	1.34	0.05	2296455, 2292515, 2289525, 2299912	113	24	3152	4.65	0.99	0.52	43.16
	SP_PIR_KEYWORDS	cell junction	4	1.34	0.05	2296455, 2292515, 2289525, 2299912	113	24	3152	4.65	0.99	0.52	43.16
	GOTERM_CC_FAT	GO:0044459~plasma membrane part	7	2.35	0.05	2294482, 2296455, 2292515, 2295347, 2289525, 2295407, 2299912	45	83	1317	2.47	0.92	0.34	40.35
	GOTERM_CC_FAT	GO:0030054~cell junction	5	1.68	0.06	2296455, 2292515, 2289525, 2295407, 2299912	45	44	1317	3.33	0.92	0.31	41.61
	GOTERM_CC_FAT	GO:0044456~synapse part	5	1.68	0.08	2293300, 2296455, 2292515, 2289525, 2299912	45	49	1317	2.99	0.97	0.36	52.91
	GOTERM_CC_FAT	GO:0045202~synapse	5	1.68	0.08	2293300, 2296455, 2292515, 2289525, 2299912	45	49	1317	2.99	0.97	0.36	52.91
	SP_PIR_KEYWORDS	membrane	14	4.70	0.08	2296588, 2293786, 2297259, 2296455, 2294910, 2295322, 2297956, 2292515, 2298009, 2291073, 2289525, 2290960, 2299912, 2298638	113	239	3152	1.63	1.00	0.57	58.28
	INTERPRO	IPR006028:Gamma-aminobutyric acid A receptor	3	1.01	0.08	2296455, 2292515, 2299912	251	14	7207	6.15	1.00	0.78	70.18
	GOTERM_MF_FAT	GO:0022836~gated channel activity	6	2.01	0.09	2293300, 2296455, 2292515, 2295347, 2289525, 2299912	137	71	4047	2.50	1.00	0.75	69.09
	GOTERM_MF_FAT	GO:0022803~passive transmembrane transporter activity	7	2.35	0.16	2296588, 2293300, 2296455, 2292515, 2295347, 2289525, 2299912	137	110	4047	1.88	1.00	0.90	89.49
	GOTERM_MF_FAT	GO:0015267~channel activity	7	2.35	0.16	2296588, 2293300, 2296455, 2292515, 2295347, 2289525, 2299912	137	110	4047	1.88	1.00	0.90	89.49
	GOTERM_MF_FAT	GO:0022838~substrate specific channel activity	7	2.35	0.16	2296588, 2293300, 2296455, 2292515, 2295347, 2289525, 2299912	137	110	4047	1.88	1.00	0.90	89.49

	GOTERM_MF_FAT	GO:0005216~ion channel activity	7	2.35	0.16	2296588, 2293300, 2296455, 2292515, 2295347, 2289525, 2299912	137	110	4047	1.88	1.00	0.90	89.49
	SP_PIR_KEYWORDS	ionic channel	6	2.01	0.18	2296588, 2296455, 2292515, 2295347, 2289525, 2299912	113	84	3152	1.99	1.00	0.80	87.53
	SP_PIR_KEYWORDS	ion transport	6	2.01	0.18	2296588, 2296455, 2292515, 2295347, 2289525, 2299912	113	85	3152	1.97	1.00	0.78	88.48
	SP_PIR_KEYWORDS	transport	9	3.02	0.24	2296588, 2293165, 2296455, 2292515, 2295347, 2291073, 2289525, 2299912, 2297692	113	167	3152	1.50	1.00	0.80	94.72
	GOTERM_BP_FAT	GO:0006811~ion transport	8	2.68	0.45	2296588, 2293300, 2296455, 2292515, 2295347, 2292188, 2289525, 2299912	94	193	2832	1.25	1.00	1.00	99.94
	GOTERM_MF_FAT	GO:0046873~metal ion transmembrane transporter activity	4	1.34	0.46	2296588, 2295347, 2292188, 2289525	137	74	4047	1.60	1.00	1.00	99.95
	GOTERM_MF_FAT	GO:0005261~cation channel activity	3	1.01	0.57	2296588, 2295347, 2289525	137	56	4047	1.58	1.00	1.00	100.00
	GOTERM_BP_FAT	GO:0030001~metal ion transport	3	1.01	0.70	2296588, 2295347, 2292188	94	73	2832	1.24	1.00	1.00	100.00
	GOTERM_BP_FAT	GO:0006812~cation transport	3	1.01	0.93	2296588, 2295347, 2292188	94	130	2832	0.70	1.00	1.00	100.00
Annotation Cluster 10	GOTERM_MF_FAT	GO:0030414~peptidase inhibitor activity	5	1.68	0.05	2289977, 2296546, 2294353, 2295831, 2290790	137	43	4047	3.43	1.00	0.73	50.88
Enrichment Score: 0.92	GOTERM_MF_FAT	GO:0004857~enzyme inhibitor activity	5	1.68	0.08	2289977, 2296546, 2294353, 2295831, 2290790	137	49	4047	3.01	1.00	0.77	65.47
	GOTERM_MF_FAT	GO:0004866~endopeptidase inhibitor activity	3	1.01	0.39	2289977, 2294353, 2290790	137	40	4047	2.22	1.00	0.99	99.81
Annotation Cluster 11	INTERPRO	IPR001313:Pumilio RNA-binding region	3	1.01	0.02	2297148, 2291717, 2290052	251	6	7207	14.36	1.00	0.43	20.63
Enrichment Score: 0.82	SMART	SM00025:Pumilio	3	1.01	0.02	2297148, 2291717, 2290052	121	6	3277	13.54	0.84	0.60	18.33
	COG_ONTOLOGY	Translation, ribosomal structure and biogenesis	3	1.01	0.51	2297148, 2291717, 2290052	26	48	722	1.74	1.00	0.99	99.06
	INTERPRO	IPR011989:Armadillo-like helical	3	1.01	0.57	2297148, 2291717, 2290052	251	55	7207	1.57	1.00	1.00	100.00
	GOTERM_MF_FAT	GO:0003723~RNA binding	4	1.34	0.90	2297148, 2291717, 2290052, 2295604	137	153	4047	0.77	1.00	1.00	100.00
Annotation Cluster 12	PIR_SUPERFAMILY	PIRSF038285:cuticle collagen	4	1.34	0.04	2298208, 2292335, 2293317, 2291120	37	24	1091	4.91	0.80	0.80	31.81
Enrichment Score: 0.79	GOTERM_MF_FAT	GO:0042302~structural constituent of cuticle	5	1.68	0.06	2298208, 2292335, 2293317, 2295005, 2291120	137	44	4047	3.36	1.00	0.68	53.42
	INTERPRO	IPR002486:Nematode cuticle collagen, N-terminal	5	1.68	0.07	2298208, 2292335, 2293317, 2295005, 2291120	251	44	7207	3.26	1.00	0.71	60.94
	SP_PIR_KEYWORDS	collagen	5	1.68	0.33	2298208, 2292335, 2293317, 2295005, 2291120	113	82	3152	1.70	1.00	0.88	98.69
	INTERPRO	IPR008160:Collagen triple helix repeat	5	1.68	0.36	2298208, 2292335, 2293317, 2295005, 2291120	251	88	7207	1.63	1.00	0.99	99.82
	GOTERM_MF_FAT	GO:0005198~structural molecule activity	6	2.01	0.92	2298208, 2295292, 2292335, 2293317, 2295005, 2291120	137	244	4047	0.73	1.00	1.00	100.00
Annotation Cluster 13	INTERPRO	IPR003591:Leucine-rich repeat, typical subtype	3	1.01	0.12	2291211, 2296553, 2298134	251	17	7207	5.07	1.00	0.88	82.13
Enrichment Score: 0.73	SMART	SM00369:LRR_TYP	3	1.01	0.13	2291211, 2296553, 2298134	121	17	3277	4.78	1.00	0.89	77.67
	SP_PIR_KEYWORDS	leucine-rich repeat	3	1.01	0.21	2291211, 2296553, 2298134	113	24	3152	3.49	1.00	0.78	91.88
	INTERPRO	IPR001611:Leucine-rich repeat	3	1.01	0.41	2291211, 2296553, 2298134	251	40	7207	2.15	1.00	1.00	99.93
Annotation Cluster 14	GOTERM_BP_FAT	GO:0045333~cellular respiration	3	1.01	0.21	2290949, 2290321, 2296921	94	26	2832	3.48	1.00	1.00	94.44
Enrichment Score: 0.51	GOTERM_BP_FAT	GO:0015980~energy derivation by oxidation of organic compounds	3	1.01	0.33	2290949, 2290321, 2296921	94	36	2832	2.51	1.00	1.00	99.30
	GOTERM_BP_FAT	GO:0055114~oxidation reduction	7	2.35	0.33	2294523, 2290949, 2291984, 2290321, 2296921, 2299186, 2296554	94	144	2832	1.46	1.00	1.00	99.33
	GOTERM_BP_FAT	GO:0006091~generation of precursor metabolites and energy	5	1.68	0.40	2294523, 2290949, 2290321, 2296921, 2300301	94	98	2832	1.54	1.00	1.00	99.82
Annotation Cluster 15	KEGG_PATHWAY	bmy00010:Glycolysis / Gluconeogenesis	3	1.01	0.08	2294523, 2298573, 2300301	21	24	1042	6.20	0.92	0.92	49.39
Enrichment Score: 0.49	GOTERM_BP_FAT	GO:0006006~glucose metabolic process	3	1.01	0.47	2294523, 2298573, 2300301	94	48	2832	1.88	1.00	1.00	99.96
	GOTERM_BP_FAT	GO:0005996~monosaccharide metabolic process	3	1.01	0.56	2294523, 2298573, 2300301	94	57	2832	1.59	1.00	1.00	100.00
	GOTERM_BP_FAT	GO:0019318~hexose metabolic process	3	1.01	0.56	2294523, 2298573, 2300301	94	57	2832	1.59	1.00	1.00	100.00
Annotation Cluster 16	GOTERM_MF_FAT	GO:0003707~steroid hormone receptor activity	4	1.34	0.08	2295281, 2291641, 2298196, 2289471	137	31	4047	3.81	1.00	0.76	67.05
Enrichment Score: 0.44	GOTERM_MF_FAT	GO:0004879~ligand-dependent nuclear receptor activity	4	1.34	0.11	2295281, 2291641, 2298196, 2289471	137	35	4047	3.38	1.00	0.81	77.50
	INTERPRO	IPR001723:Steroid hormone receptor	3	1.01	0.16	2291641, 2298196, 2289471	251	21	7207	4.10	1.00	0.93	91.80
	INTERPRO	IPR000324:Vitamin D receptor	3	1.01	0.18	2295281, 2291641, 2298196	251	22	7207	3.92	1.00	0.93	93.36
	INTERPRO	IPR008946:Nuclear hormone receptor, ligand-binding	3	1.01	0.25	2291641, 2298196, 2289471	251	28	7207	3.08	1.00	0.97	98.32
	INTERPRO	IPR000536:Nuclear hormone receptor, ligand-binding, core	3	1.01	0.27	2291641, 2298196, 2289471	251	29	7207	2.97	1.00	0.98	98.68
	SMART	SM00430:HOLI	3	1.01	0.29	2291641, 2298196, 2289471	121	29	3277	2.80	1.00	0.95	97.66
	GOTERM_MF_FAT	GO:0030528~transcription regulator activity	12	4.03	0.33	2295593, 2295220, 2293305, 2292026, 2291938, 2295281, 2299992, 2291641, 2298196, 2298660, 2293102, 2289471	137	278	4047	1.28	1.00	0.99	99.37
	INTERPRO	IPR001628:Zinc finger, nuclear hormone receptor-type	3	1.01	0.34	2295281, 2291641, 2298196	251	35	7207	2.46	1.00	0.99	99.72
	GOTERM_BP_FAT	GO:0045449~regulation of transcription	13	4.36	0.35	2295220, 2291934, 2293305, 2292026, 2291938, 2295281, 2299992, 2289494, 2291641, 2298196, 2298660, 2293102, 2289471	94	319	2832	1.23	1.00	1.00	99.53
	SMART	SM00399:ZnF_C4	3	1.01	0.37	2295281, 2291641, 2298196	121	35	3277	2.32	1.00	0.97	99.39
	INTERPRO	IPR013088:Zinc finger, NHR/GATA-type	3	1.01	0.38	2295281, 2291641, 2298196	251	38	7207	2.27	1.00	0.99	99.88
	GOTERM_MF_FAT	GO:0003700~transcription factor activity	8	2.68	0.41	2295220, 2291938, 2295281, 2299992, 2291641, 2298196, 2298660, 2289471	137	181	4047	1.31	1.00	0.99	99.86
	SP_PIR_KEYWORDS	transcription regulation	4	1.34	0.51	2295281, 2291641, 2298196, 2289471	113	76	3152	1.47	1.00	0.96	99.95
	GOTERM_BP_FAT	GO:0006350~transcription	5	1.68	0.52	2291934, 2295281, 2291641, 2298196, 2289471	94	114	2832	1.32	1.00	1.00	99.99
	GOTERM_MF_FAT	GO:0043565~sequence-specific DNA binding	6	2.01	0.54	2291938, 2295281, 2299992, 2291641, 2298196, 2298660	137	144	4047	1.23	1.00	1.00	99.99
	SP_PIR_KEYWORDS	Transcription	4	1.34	0.58	2295281, 2291641, 2298196, 2289471	113	84	3152	1.33	1.00	0.97	99.99
	GOTERM_BP_FAT	GO:0006355~regulation of transcription, DNA-dependent	8	2.68	0.67	2295220, 2291938, 2295281, 2299992, 2291641, 2298196, 2298660, 2289471	94	236	2832	1.02	1.00	1.00	100.00
	GOTERM_BP_FAT	GO:0051252~regulation of RNA metabolic process	8	2.68	0.67	2295220, 2291938, 2295281, 2299992, 2291641, 2298196, 2298660, 2289471	94	236	2832	1.02	1.00	1.00	100.00
	SP_PIR_KEYWORDS	dna-binding	8	2.68	0.72	2298392, 2293305, 2292026, 2295281, 2299992, 2291641, 2298196, 2298660	113	230	3152	0.97	1.00	0.99	100.00
	GOTERM_MF_FAT	GO:0003677~DNA binding	16	5.37	0.77	2295220, 2293305, 2292026, 2299992, 2289494, 2291641, 2293405, 2298660, 2293102, 2289471, 2291934, 2298392, 2295281, 2291938, 2298196, 2299106	137	519	4047	0.91	1.00	1.00	100.00
	SP_PIR_KEYWORDS	nucleus	7	2.35	0.85	2298392, 2295281, 2299992, 2291641, 2298196, 2298660, 2289471	113	232	3152	0.84	1.00	1.00	100.00
Annotation Cluster 17	GOTERM_BP_FAT	GO:0051056~regulation of small GTPase mediated signal transduction	4	1.34	0.34	2297565, 2297615, 2298950, 2290362	94	63	2832	1.91	1.00	1.00	99.42

Enrichment Score: 0.36	GOTERM_MF_FAT	GO:0030695~GTPase regulator activity	4	1.34	0.49	2297565, 2297615, 2298950, 2290362	137	78	4047	1.51	1.00	1.00	99.98
	GOTERM_MF_FAT	GO:0060589~nucleoside-triphosphatase regulator activity	4	1.34	0.51	2297565, 2297615, 2298950, 2290362	137	80	4047	1.48	1.00	1.00	99.99
Annotation Cluster 18	GOTERM_MF_FAT	GO:0004725~protein tyrosine phosphatase activity	3	1.01	0.56	2289670, 2297696, 2290856	137	55	4047	1.61	1.00	1.00	100.00
Enrichment Score: 0.19	GOTERM_BP_FAT	GO:0006470~protein amino acid dephosphorylation	3	1.01	0.61	2289670, 2297696, 2290856	94	62	2832	1.46	1.00	1.00	100.00
	GOTERM_MF_FAT	GO:0004721~phosphoprotein phosphatase activity	4	1.34	0.63	2289670, 2291511, 2297696, 2290856	137	95	4047	1.24	1.00	1.00	100.00
	GOTERM_BP_FAT	GO:0016311~dephosphorylation	3	1.01	0.68	2289670, 2297696, 2290856	94	70	2832	1.29	1.00	1.00	100.00
	GOTERM_MF_FAT	GO:0016791~phosphatase activity	4	1.34	0.81	2289670, 2291511, 2297696, 2290856	137	128	4047	0.92	1.00	1.00	100.00
Annotation Cluster 19	SP_PIR_KEYWORDS	ank repeat	3	1.01	0.66	2298367, 2294841, 2294741	113	63	3152	1.33	1.00	0.99	100.00
Enrichment Score: 0.16	INTERPRO	IPR002110:Ankyrin	3	1.01	0.70	2298367, 2294841, 2294741	251	69	7207	1.25	1.00	1.00	100.00
	SMART	SM00248:ANK	3	1.01	0.73	2298367, 2294841, 2294741	121	69	3277	1.18	1.00	1.00	100.00
Annotation Cluster 20	SP_PIR_KEYWORDS	metal-binding	14	4.70	0.34	2291984, 2299522, 2294308, 2291641, 2300095, 2297553, 2291795, 2298280, 2295209, 2297330, 2295281, 2298196, 2298171, 2293642	113	318	3152	1.23	1.00	0.87	98.80
Enrichment Score: 0.15	SP_PIR_KEYWORDS	zinc	11	3.69	0.52	2295209, 2294308, 2299522, 2297330, 2295281, 2291641, 2300095, 2298196, 2293642, 2297553, 2291795	113	276	3152	1.11	1.00	0.96	99.96
	GOTERM_MF_FAT	GO:0043169~cation binding	32	10.74	0.83	2298048, 2291984, 2298009, 2294720, 2296422, 2290176, 2291795, 2298638, 2295209, 2295281, 2295347, 2292414, 2298196, 2300013, 2293950, 2293851, 2291857, 2294308, 2299522, 2299149, 2293303, 2291641, 2299491, 2290446, 2294454, 2300095, 2296172, 2297553, 2293547, 2297330, 2300057, 2293642	137	1050	4047	0.90	1.00	1.00	100.00
	GOTERM_MF_FAT	GO:0043167~ion binding	32	10.74	0.83	2298048, 2291984, 2298009, 2294720, 2296422, 2290176, 2291795, 2298638, 2295209, 2295281, 2295347, 2292414, 2298196, 2300013, 2293950, 2293851, 2291857, 2294308, 2299522, 2299149, 2293303, 2291641, 2299491, 2290446, 2294454, 2300095, 2296172, 2297553, 2293547, 2297330, 2300057, 2293642	137	1050	4047	0.90	1.00	1.00	100.00
	GOTERM_MF_FAT	GO:0046872~metal ion binding	31	10.40	0.85	2291984, 2298009, 2294720, 2296422, 2290176, 2291795, 2298638, 2295209, 2295281, 2295347, 2292414, 2298196, 2300013, 2293950, 2293851, 2291857, 2294308, 2299522, 2299149, 2293303, 2291641, 2299491, 2290446, 2294454, 2300095, 2296172, 2297553, 2293547, 2297330, 2300057, 2293642	137	1032	4047	0.89	1.00	1.00	100.00
	GOTERM_MF_FAT	GO:0008270~zinc ion binding	20	6.71	0.91	2293950, 2294308, 2299522, 2293303, 2299149, 2291641, 2299491, 2300095, 2294720, 2297553, 2291795, 2290176, 2293547, 2295209, 2295281, 2297330, 2292414, 2300057, 2298196, 2293642	137	725	4047	0.81	1.00	1.00	100.00
	GOTERM_MF_FAT	GO:0046914~transition metal ion binding	23	7.72	0.92	2293950, 2300013, 2291984, 2294308, 2299522, 2293303, 2299149, 2291641, 2299491, 2300095, 2296172, 2294720, 2297553, 2290176, 2291795, 2293547, 2295209, 2295281, 2297330, 2292414, 2300057, 2298196, 2293642	137	833	4047	0.82	1.00	1.00	100.00
Annotation Cluster 21	INTERPRO	IPR001245:Tyrosine protein kinase	4	1.34	0.31	2299418, 2291320, 2298992, 2290789	251	56	7207	2.05	1.00	0.99	99.41
Enrichment Score: 0.10	SMART	SM00219:TyrKc	4	1.34	0.34	2299418, 2291320, 2298992, 2290789	121	56	3277	1.93	1.00	0.97	98.95
	GOTERM_MF_FAT	GO:0004713~protein tyrosine kinase activity	4	1.34	0.35	2299418, 2291320, 2298992, 2290789	137	62	4047	1.91	1.00	0.99	99.53
	GOTERM_BP_FAT	GO:0006793~phosphorus metabolic process	12	4.03	0.75	2289670, 2299418, 2299130, 2290949, 2297082, 2294308, 2296921, 2291320, 2297696, 2298992, 2290789, 2290856	94	390	2832	0.93	1.00	1.00	100.00
	GOTERM_BP_FAT	GO:0006796~phosphate metabolic process	12	4.03	0.75	2289670, 2299418, 2299130, 2290949, 2297082, 2294308, 2296921, 2291320, 2297696, 2298992, 2290789, 2290856	94	390	2832	0.93	1.00	1.00	100.00
	GOTERM_BP_FAT	GO:0016310~phosphorylation	8	2.68	0.91	2299418, 2290949, 2297082, 2294308, 2296921, 2291320, 2298992, 2290789	94	315	2832	0.77	1.00	1.00	100.00
	SP_PIR_KEYWORDS	kinase	9	3.02	0.92	2299418, 2299130, 2297082, 2294308, 2290748, 2296758, 2289458, 2290789, 2293683	113	332	3152	0.76	1.00	1.00	100.00
	GOTERM_BP_FAT	GO:0006468~protein amino acid phosphorylation	6	2.01	0.95	2299418, 2297082, 2294308, 2291320, 2298992, 2290789	94	265	2832	0.68	1.00	1.00	100.00
	GOTERM_MF_FAT	GO:0004672~protein kinase activity	6	2.01	0.96	2299418, 2297082, 2294308, 2291320, 2298992, 2290789	137	271	4047	0.65	1.00	1.00	100.00
	INTERPRO	IPR000719:Protein kinase, core	5	1.68	0.98	2299418, 2297082, 2294308, 2291320, 2298992	251	251	7207	0.57	1.00	1.00	100.00
	GOTERM_MF_FAT	GO:0005524~ATP binding	11	3.69	1.00	2291958, 2299418, 2297082, 2294308, 2291320, 2298992, 2293405, 2289458, 2296937, 2290789, 2299106	137	629	4047	0.52	1.00	1.00	100.00
	GOTERM_MF_FAT	GO:0032559~adenyl ribonucleotide binding	11	3.69	1.00	2291958, 2299418, 2297082, 2294308, 2291320, 2298992, 2293405, 2289458, 2296937, 2290789, 2299106	137	630	4047	0.52	1.00	1.00	100.00
	GOTERM_MF_FAT	GO:0001883~purine nucleoside binding	11	3.69	1.00	2291958, 2299418, 2297082, 2294308, 2291320, 2298992, 2293405, 2289458, 2296937, 2290789, 2299106	137	660	4047	0.49	1.00	1.00	100.00
	GOTERM_MF_FAT	GO:0030554~adenyl nucleotide binding	11	3.69	1.00	2291958, 2299418, 2297082, 2294308, 2291320, 2298992, 2293405, 2289458, 2296937, 2290789, 2299106	137	660	4047	0.49	1.00	1.00	100.00
	GOTERM_MF_FAT	GO:0001882~nucleoside binding	11	3.69	1.00	2291958, 2299418, 2297082, 2294308, 2291320, 2298992, 2293405, 2289458, 2296937, 2290789, 2299106	137	664	4047	0.49	1.00	1.00	100.00
	GOTERM_MF_FAT	GO:0032553~ribonucleotide binding	12	4.03	1.00	2291958, 2299418, 2297082, 2294308, 2291320, 2298992, 2293405, 2289458, 2296937, 2290789, 2290960, 2299106	137	770	4047	0.46	1.00	1.00	100.00
	GOTERM_MF_FAT	GO:0032555~purine ribonucleotide binding	12	4.03	1.00	2291958, 2299418, 2297082, 2294308, 2291320, 2298992, 2293405, 2289458, 2296937, 2290789, 2290960, 2299106	137	770	4047	0.46	1.00	1.00	100.00
	GOTERM_MF_FAT	GO:0000166~nucleotide binding	16	5.37	1.00	2294308, 2291320, 2293405, 2289458, 2290789, 2296937, 2299418, 2291958, 2300269, 2291761, 2298573, 2297082, 2298992, 2290960, 2293935, 2299106	137	963	4047	0.49	1.00	1.00	100.00
	GOTERM_MF_FAT	GO:0017076~purine nucleotide binding	12	4.03	1.00	2291958, 2299418, 2297082, 2294308, 2291320, 2298992, 2293405, 2289458, 2296937, 2290789, 2290960, 2299106	137	806	4047	0.44	1.00	1.00	100.00
Annotation Cluster 22	INTERPRO	IPR013087:Zinc finger, C2H2-type/integrase, DNA-binding	3	1.01	0.69	2293950, 2299149, 2294720	251	68	7207	1.27	1.00	1.00	100.00
Enrichment Score: 0.05	INTERPRO	IPR007087:Zinc finger, C2H2-type	5	1.68	0.98	2293950, 2293303, 2299149, 2300057, 2294720	251	256	7207	0.56	1.00	1.00	100.00
	INTERPRO	IPR015880:Zinc finger, C2H2-like	5	1.68	0.98	2293950, 2293303, 2299149, 2300057, 2294720	251	266	7207	0.54	1.00	1.00	100.00
	SMART	SM00355:ZnF_C2H2	5	1.68	0.99	2293950, 2293303, 2299149, 2300057, 2294720	121	266	3277	0.51	1.00	1.00	100.00
Annotation Cluster 23	INTERPRO	IPR012677:Nucleotide-binding, alpha-beta plait	3	1.01	0.91	2300269, 2291761, 2293935	251	113	7207	0.76	1.00	1.00	100.00
Enrichment Score: 0.04	INTERPRO	IPR000504:RNA recognition motif, RNP-1	3	1.01	0.91	2300269, 2291761, 2293935	251	115	7207	0.75	1.00	1.00	100.00

	SMART	SM00360:RRM	3	1.01	0.93	2300269, 2291761, 2293935	121	115	3277	0.71	1.00	1.00	100.00
Annotation Cluster 24	GOTERM_CC_FAT	GO:0005856~cytoskeleton	3	1.01	0.95	2294983, 2296937, 2298581	45	135	1317	0.65	1.00	1.00	100.00
Enrichment Score: 0.01	GOTERM_CC_FAT	GO:0043228~non-membrane-bounded organelle	5	1.68	1.00	2298392, 2295292, 2294983, 2296937, 2298581	45	347	1317	0.42	1.00	1.00	100.00
	GOTERM_CC_FAT	GO:0043232~intracellular non-membrane-bounded organelle	5	1.68	1.00	2298392, 2295292, 2294983, 2296937, 2298581	45	347	1317	0.42	1.00	1.00	100.00

24-h *B. a.*

Annotation Cluster 1	INTERPRO	IPR000742:EGF-like, type 3	11	6.36	0.00	2289402, 2293903, 2296386, 2291857, 2292811, 2294910, 2300247, 2294112, 2294454, 2290446, 2294634	147	56	7207	9.63	0.00	0.00	0.00
Enrichment Score: 4.61	INTERPRO	IPR006210:EGF-like	10	5.78	0.00	2289402, 2293903, 2296386, 2291857, 2292811, 2294910, 2300247, 2294112, 2294454, 2294634	147	69	7207	7.11	0.00	0.00	0.01
	SP_PIR_KEYWORDS	egf-like domain	9	5.20	0.00	2289402, 2296386, 2291857, 2292811, 2294910, 2300247, 2294112, 2294454, 2290446	65	57	3152	7.66	0.00	0.00	0.01
	INTERPRO	IPR013032:EGF-like region, conserved site	11	6.36	0.00	2293903, 2294482, 2296386, 2291857, 2292811, 2294910, 2300247, 2294454, 2290446, 2300095, 2294634	147	92	7207	5.86	0.00	0.00	0.02
	SMART	SM00181:EGF	10	5.78	0.00	2289402, 2293903, 2296386, 2291857, 2292811, 2294910, 2300247, 2294112, 2294454, 2294634	74	69	3277	6.42	0.00	0.00	0.02
	INTERPRO	IPR000152:EGF-type aspartate/asparagine hydroxylation conserved site	6	3.47	0.00	2289402, 2296386, 2291857, 2294112, 2294454, 2290446	147	34	7207	8.65	0.13	0.03	0.71
	INTERPRO	IPR006209:EGF	5	2.89	0.00	2293903, 2291857, 2300247, 2294112, 2294454	147	28	7207	8.75	0.43	0.09	2.90
Annotation Cluster 2	GOTERM_BP_FAT	GO:0007155~cell adhesion	5	2.89	0.01	2299418, 2294482, 2296386, 2298009, 2290446	53	43	2832	6.21	0.58	0.58	8.10
Enrichment Score: 2.09	GOTERM_BP_FAT	GO:0022610~biological adhesion	5	2.89	0.01	2299418, 2294482, 2296386, 2298009, 2290446	53	43	2832	6.21	0.58	0.58	8.10
	GOTERM_BP_FAT	GO:0007160~cell-matrix adhesion	3	1.73	0.01	2294482, 2296386, 2290446	53	8	2832	20.04	0.64	0.40	9.44
	GOTERM_BP_FAT	GO:0031589~cell-substrate adhesion	3	1.73	0.01	2294482, 2296386, 2290446	53	8	2832	20.04	0.64	0.40	9.44
Annotation Cluster 3	INTERPRO	IPR000152:EGF-type aspartate/asparagine hydroxylation conserved site	6	3.47	0.00	2289402, 2296386, 2291857, 2294112, 2294454, 2290446	147	34	7207	8.65	0.13	0.03	0.71
Enrichment Score: 1.51	GOTERM_MF_FAT	GO:0005509~calcium ion binding	7	4.05	0.04	2293851, 2295990, 2291857, 2298009, 2295347, 2294454, 2290446	78	137	4047	2.65	1.00	0.97	42.32
	INTERPRO	IPR018097:EGF-like calcium-binding, conserved site	3	1.73	0.09	2291857, 2294454, 2290446	147	25	7207	5.88	1.00	0.84	70.77
	INTERPRO	IPR001881:EGF-like calcium-binding	3	1.73	0.10	2291857, 2294454, 2290446	147	27	7207	5.45	1.00	0.84	75.63
	SMART	SM00179:EGF_CA	3	1.73	0.12	2291857, 2294454, 2290446	74	27	3277	4.92	1.00	0.83	73.25
Annotation Cluster 4	GOTERM_CC_FAT	GO:0016021~integral to membrane	20	11.56	0.00	2300676, 2293786, 2290374, 2294482, 2295322, 2289525, 2297692, 2296588, 2291072, 2295396, 2294159, 2293165, 2296455, 2297956, 2291073, 2295347, 2296494, 2297177, 2299912, 2294340	29	519	1317	1.75	0.06	0.06	1.72
Enrichment Score: 1.09	GOTERM_CC_FAT	GO:0031224~intrinsic to membrane	20	11.56	0.00	2300676, 2293786, 2290374, 2294482, 2295322, 2289525, 2297692, 2296588, 2291072, 2295396, 2294159, 2293165, 2296455, 2297956, 2291073, 2295347, 2296494, 2297177, 2299912, 2294340	29	527	1317	1.72	0.08	0.04	2.13
	SP_PIR_KEYWORDS	transmembrane	14	8.09	0.01	2293786, 2290374, 2295322, 2298009, 2289525, 2297692, 2296588, 2293165, 2296455, 2297956, 2291073, 2295347, 2299912, 2294340	65	311	3152	2.18	0.39	0.22	7.97
	GOTERM_MF_FAT	GO:0030594~neurotransmitter receptor activity	4	2.31	0.02	2290374, 2296455, 2289525, 2299912	78	29	4047	7.16	0.93	0.93	18.55
	GOTERM_MF_FAT	GO:0042165~neurotransmitter binding	4	2.31	0.02	2290374, 2296455, 2289525, 2299912	78	29	4047	7.16	0.93	0.93	18.55
	GOTERM_CC_FAT	GO:0044459~plasma membrane part	6	3.47	0.03	2294482, 2296455, 2295347, 2289525, 2295407, 2299912	29	83	1317	3.28	0.61	0.21	21.69
	GOTERM_CC_FAT	GO:0005886~plasma membrane	6	3.47	0.04	2294482, 2296455, 2295347, 2289525, 2295407, 2299912	29	91	1317	2.99	0.74	0.24	29.51
	SP_PIR_KEYWORDS	membrane	10	5.78	0.05	2296588, 2293786, 2296455, 2294910, 2295322, 2297956, 2298009, 2291073, 2289525, 2299912	65	239	3152	2.03	0.95	0.64	40.05
	SP_PIR_KEYWORDS	transport	8	4.62	0.05	2296588, 2293165, 2296455, 2291073, 2295347, 2289525, 2299912, 2297692	65	167	3152	2.32	0.95	0.54	40.41
	GOTERM_CC_FAT	GO:0030054~cell junction	4	2.31	0.06	2296455, 2289525, 2295407, 2299912	29	44	1317	4.13	0.89	0.31	43.94
	INTERPRO	IPR018000:Neurotransmitter-gated ion-channel, conserved site	3	1.73	0.07	2296455, 2289525, 2299912	147	22	7207	6.69	1.00	0.82	62.28
	SP_PIR_KEYWORDS	postsynaptic cell membrane	3	1.73	0.08	2296455, 2289525, 2299912	65	23	3152	6.33	0.99	0.56	55.76
	SP_PIR_KEYWORDS	cell junction	3	1.73	0.08	2296455, 2289525, 2299912	65	24	3152	6.06	0.99	0.53	58.57
	SP_PIR_KEYWORDS	synapse	3	1.73	0.08	2296455, 2289525, 2299912	65	24	3152	6.06	0.99	0.53	58.57
	INTERPRO	IPR006029:Neurotransmitter-gated ion-channel transmembrane region	3	1.73	0.08	2296455, 2289525, 2299912	147	24	7207	6.13	1.00	0.84	68.09
	SP_PIR_KEYWORDS	ionic channel	5	2.89	0.09	2296588, 2296455, 2295347, 2289525, 2299912	65	84	3152	2.89	1.00	0.50	60.86
	SP_PIR_KEYWORDS	ion transport	5	2.89	0.09	2296588, 2296455, 2295347, 2289525, 2299912	65	85	3152	2.85	1.00	0.48	62.15
	SP_PIR_KEYWORDS	cell membrane	3	1.73	0.13	2296455, 2289525, 2299912	65	31	3152	4.69	1.00	0.57	75.16
	INTERPRO	IPR006202:Neurotransmitter-gated ion-channel ligand-binding	3	1.73	0.14	2296455, 2289525, 2299912	147	32	7207	4.60	1.00	0.90	85.14
	GOTERM_MF_FAT	GO:0022836~gated channel activity	4	2.31	0.15	2296455, 2295347, 2289525, 2299912	78	71	4047	2.92	1.00	0.90	86.13
	GOTERM_MF_FAT	GO:0005216~ion channel activity	5	2.89	0.16	2296588, 2296455, 2295347, 2289525, 2299912	78	110	4047	2.36	1.00	0.89	86.91
	GOTERM_MF_FAT	GO:0015267~channel activity	5	2.89	0.16	2296588, 2296455, 2295347, 2289525, 2299912	78	110	4047	2.36	1.00	0.89	86.91
	GOTERM_MF_FAT	GO:0022803~passive transmembrane transporter activity	5	2.89	0.16	2296588, 2296455, 2295347, 2289525, 2299912	78	110	4047	2.36	1.00	0.89	86.91
	GOTERM_MF_FAT	GO:0022838~substrate specific channel activity	5	2.89	0.16	2296588, 2296455, 2295347, 2289525, 2299912	78	110	4047	2.36	1.00	0.89	86.91
	GOTERM_MF_FAT	GO:0005230~extracellular ligand-gated ion channel activity	3	1.73	0.18	2296455, 2289525, 2299912	78	40	4047	3.89	1.00	0.90	90.19
	INTERPRO	IPR006201:Neurotransmitter-gated ion-channel	3	1.73	0.18	2296455, 2289525, 2299912	147	38	7207	3.87	1.00	0.95	92.32
	GOTERM_MF_FAT	GO:0022834~ligand-gated channel activity	3	1.73	0.19	2296455, 2289525, 2299912	78	42	4047	3.71	1.00	0.88	91.99
	GOTERM_MF_FAT	GO:0015276~ligand-gated ion channel activity	3	1.73	0.19	2296455, 2289525, 2299912	78	42	4047	3.71	1.00	0.88	91.99
	GOTERM_CC_FAT	GO:0045211~postsynaptic membrane	3	1.73	0.22	2296455, 2289525, 2299912	29	41	1317	3.32	1.00	0.69	88.20
	GOTERM_CC_FAT	GO:0044456~synapse part	3	1.73	0.28	2296455, 2289525, 2299912	29	49	1317	2.78	1.00	0.75	94.43
	GOTERM_CC_FAT	GO:0045202~synapse	3	1.73	0.28	2296455, 2289525, 2299912	29	49	1317	2.78	1.00	0.75	94.43
	GOTERM_MF_FAT	GO:0005261~cation channel activity	3	1.73	0.29	2296588, 2295347, 2289525	78	56	4047	2.78	1.00	0.95	98.32
	GOTERM_MF_FAT	GO:0046873~metal ion transmembrane transporter activity	3	1.73	0.41	2296588, 2295347, 2289525	78	74	4047	2.10	1.00	0.99	99.83
	GOTERM_BP_FAT	GO:0006811~ion transport	5	2.89	0.48	2296588, 2296455, 2295347, 2289525, 2299912	53	193	2832	1.38	1.00	1.00	99.94

Annotation Cluster 5	INTERPRO	IPR000731:Sterol-sensing 5TM box	3	1.73	0.08	2295396, 2300676, 2294159	147	24	7207	6.13	1.00	0.84	68.09
Enrichment Score: 1.05	GOTERM_MF_FAT	GO:0008158~hedgehog receptor activity	3	1.73	0.09	2295396, 2300676, 2294159	78	26	4047	5.99	1.00	0.79	66.26
	INTERPRO	IPR003392:Patched	3	1.73	0.10	2295396, 2300676, 2294159	147	26	7207	5.66	1.00	0.84	73.28
Annotation Cluster 6	GOTERM_MF_FAT	GO:0008237~metallopeptidase activity	5	2.89	0.05	2293547, 2293573, 2295209, 2297765, 2300095	78	75	4047	3.46	1.00	0.88	48.20
Enrichment Score: 0.92	GOTERM_MF_FAT	GO:0070011~peptidase activity, acting on L-amino acid peptides	8	4.62	0.06	2292564, 2293547, 2293573, 2295209, 2297765, 2292649, 2300095, 2296172	78	188	4047	2.21	1.00	0.87	55.25
	GOTERM_MF_FAT	GO:0004222~metalloendopeptidase activity	4	2.31	0.07	2293573, 2295209, 2297765, 2300095	78	50	4047	4.15	1.00	0.79	57.39
	GOTERM_MF_FAT	GO:0004175~endopeptidase activity	6	3.47	0.08	2292564, 2293573, 2295209, 2297765, 2292649, 2300095	78	122	4047	2.55	1.00	0.80	63.82
	GOTERM_MF_FAT	GO:0008233~peptidase activity	8	4.62	0.12	2292564, 2293547, 2293573, 2295209, 2297765, 2292649, 2300095, 2296172	78	219	4047	1.90	1.00	0.86	78.69
	SP_PIR_KEYWORDS	Protease	5	2.89	0.19	2292564, 2295209, 2297765, 2292649, 2300095	65	112	3152	2.16	1.00	0.69	88.06
	GOTERM_BP_FAT	GO:0006508~proteolysis	7	4.05	0.29	2292564, 2293547, 2293573, 2295209, 2297765, 2292649, 2300095	53	245	2832	1.53	1.00	1.00	98.07
	SP_PIR_KEYWORDS	hydrolase	9	5.20	0.32	2289670, 2292089, 2294841, 2292564, 2295209, 2297696, 2292649, 2300095, 2298171	65	320	3152	1.36	1.00	0.83	97.96
Annotation Cluster 7	GOTERM_BP_FAT	GO:0051056~regulation of small GTPase mediated signal transduction	4	2.31	0.11	2297565, 2297615, 2298950, 2290362	53	63	2832	3.39	1.00	0.99	72.81
Enrichment Score: 0.80	GOTERM_MF_FAT	GO:0030695~GTPase regulator activity	4	2.31	0.18	2297565, 2297615, 2298950, 2290362	78	78	4047	2.66	1.00	0.89	91.35
	GOTERM_MF_FAT	GO:0060589~nucleoside-triphosphatase regulator activity	4	2.31	0.19	2297565, 2297615, 2298950, 2290362	78	80	4047	2.59	1.00	0.87	92.51
Annotation Cluster 8	INTERPRO	IPR013151:Immunoglobulin	3	1.73	0.08	2294830, 2300340, 2300096	147	24	7207	6.13	1.00	0.84	68.09
Enrichment Score: 0.63	INTERPRO	IPR007110:Immunoglobulin-like	3	1.73	0.39	2294830, 2300340, 2300096	147	66	7207	2.23	1.00	1.00	99.83
	INTERPRO	IPR013783:Immunoglobulin-like fold	3	1.73	0.40	2294830, 2300340, 2300096	147	67	7207	2.20	1.00	1.00	99.85
Annotation Cluster 9	GOTERM_BP_FAT	GO:0007166~cell surface receptor linked signal transduction	5	2.89	0.16	2290374, 2294482, 2295322, 2296851, 2296494	53	115	2832	2.32	1.00	0.99	86.09
Enrichment Score: 0.60	SP_PIR_KEYWORDS	receptor	6	3.47	0.22	2290374, 2295322, 2291641, 2289525, 2296494, 2298196	65	160	3152	1.82	1.00	0.72	92.02
	GOTERM_BP_FAT	GO:0007186~G-protein coupled receptor protein signaling pathway	3	1.73	0.43	2290374, 2295322, 2296494	53	79	2832	2.03	1.00	1.00	99.84
Annotation Cluster 10	GOTERM_MF_FAT	GO:0042302~structural constituent of cuticle	3	1.73	0.20	2298208, 2295005, 2291120	78	44	4047	3.54	1.00	0.87	93.49
Enrichment Score: 0.39	INTERPRO	IPR002486:Nematode cuticle collagen, N-terminal	3	1.73	0.22	2298208, 2295005, 2291120	147	44	7207	3.34	1.00	0.97	96.28
	SP_PIR_KEYWORDS	collagen	3	1.73	0.50	2298208, 2295005, 2291120	65	82	3152	1.77	1.00	0.94	99.91
	INTERPRO	IPR008160:Collagen triple helix repeat	3	1.73	0.54	2298208, 2295005, 2291120	147	88	7207	1.67	1.00	1.00	100.00
	GOTERM_MF_FAT	GO:0005198~structural molecule activity	3	1.73	0.95	2298208, 2295005, 2291120	78	244	4047	0.64	1.00	1.00	100.00
Annotation Cluster 11	GOTERM_MF_FAT	GO:0043169~cation binding	21	12.14	0.54	2298048, 2300013, 2295990, 2291857, 2293851, 2299522, 2293303, 2299149, 2298009, 2291641, 2290446, 2294454, 2300095, 2296172, 2294720, 2293547, 2295209, 2295347, 2292414, 2295025, 2298196	78	1050	4047	1.04	1.00	1.00	99.99
Enrichment Score: 0.17	GOTERM_MF_FAT	GO:0043167~ion binding	21	12.14	0.54	2298048, 2300013, 2295990, 2291857, 2293851, 2299522, 2293303, 2299149, 2298009, 2291641, 2290446, 2294454, 2300095, 2296172, 2294720, 2293547, 2295209, 2295347, 2292414, 2295025, 2298196	78	1050	4047	1.04	1.00	1.00	99.99
	GOTERM_MF_FAT	GO:0046872~metal ion binding	20	11.56	0.61	2300013, 2295990, 2291857, 2293851, 2299522, 2293303, 2299149, 2298009, 2291641, 2290446, 2294454, 2300095, 2296172, 2294720, 2293547, 2295209, 2295347, 2292414, 2295025, 2298196	78	1032	4047	1.01	1.00	1.00	100.00
	SP_PIR_KEYWORDS	metal-binding	7	4.05	0.64	2295209, 2299522, 2291641, 2295025, 2300095, 2298196, 2298171	65	318	3152	1.07	1.00	0.98	100.00
	SP_PIR_KEYWORDS	zinc	6	3.47	0.67	2295209, 2299522, 2291641, 2295025, 2300095, 2298196	65	276	3152	1.05	1.00	0.98	100.00
	GOTERM_MF_FAT	GO:0046914~transition metal ion binding	13	7.51	0.90	2300013, 2299522, 2293303, 2299149, 2291641, 2300095, 2296172, 2294720, 2293547, 2295209, 2292414, 2295025, 2298196	78	833	4047	0.81	1.00	1.00	100.00
	GOTERM_MF_FAT	GO:0008270~zinc ion binding	11	6.36	0.91	2293547, 2295209, 2299522, 2293303, 2299149, 2292414, 2291641, 2295025, 2300095, 2294720, 2298196	78	725	4047	0.79	1.00	1.00	100.00
Annotation Cluster 12	SP_PIR_KEYWORDS	dna-binding	7	4.05	0.32	2292671, 2292026, 2299817, 2291641, 2291237, 2298196, 2298660	65	230	3152	1.48	1.00	0.81	98.00
Enrichment Score: 0.15	GOTERM_MF_FAT	GO:0030528~transcription regulator activity	6	3.47	0.62	2295593, 2292026, 2291641, 2291237, 2298196, 2298660	78	278	4047	1.12	1.00	1.00	100.00
	GOTERM_BP_FAT	GO:0045449~regulation of transcription	6	3.47	0.71	2292026, 2299817, 2291641, 2291237, 2298196, 2298660	53	319	2832	1.01	1.00	1.00	100.00
	GOTERM_MF_FAT	GO:0043565~sequence-specific DNA binding	3	1.73	0.77	2291641, 2298196, 2298660	78	144	4047	1.08	1.00	1.00	100.00
	GOTERM_BP_FAT	GO:0006355~regulation of transcription, DNA-dependent	4	2.31	0.82	2299817, 2291641, 2298196, 2298660	53	236	2832	0.91	1.00	1.00	100.00
	GOTERM_BP_FAT	GO:0051252~regulation of RNA metabolic process	4	2.31	0.82	2299817, 2291641, 2298196, 2298660	53	236	2832	0.91	1.00	1.00	100.00
	SP_PIR_KEYWORDS	nucleus	4	2.31	0.86	2292671, 2291641, 2298196, 2298660	65	232	3152	0.84	1.00	1.00	100.00
	GOTERM_MF_FAT	GO:0003700~transcription factor activity	3	1.73	0.87	2291641, 2298196, 2298660	78	181	4047	0.86	1.00	1.00	100.00
	GOTERM_MF_FAT	GO:0003677~DNA binding	8	4.62	0.88	2292671, 2292026, 2299817, 2291641, 2291237, 2298196, 2298660, 2299106	78	519	4047	0.80	1.00	1.00	100.00
Annotation Cluster 13	GOTERM_BP_FAT	GO:0006793~phosphorus metabolic process	6	3.47	0.86	2289670, 2299418, 2299130, 2297082, 2296921, 2297696	53	390	2832	0.82	1.00	1.00	100.00
Enrichment Score: 0.03	GOTERM_BP_FAT	GO:0006796~phosphate metabolic process	6	3.47	0.86	2289670, 2299418, 2299130, 2297082, 2296921, 2297696	53	390	2832	0.82	1.00	1.00	100.00
	GOTERM_BP_FAT	GO:0016310~phosphorylation	3	1.73	0.98	2299418, 2297082, 2296921	53	315	2832	0.51	1.00	1.00	100.00
	SP_PIR_KEYWORDS	kinase	3	1.73	0.99	2299418, 2299130, 2297082	65	332	3152	0.44	1.00	1.00	100.00
Annotation Cluster 14	INTERPRO	IPR007087:Zinc finger, C2H2-type	3	1.73	0.97	2293303, 2299149, 2294720	147	256	7207	0.57	1.00	1.00	100.00
Enrichment Score: 0.01	INTERPRO	IPR015880:Zinc finger, C2H2-like	3	1.73	0.97	2293303, 2299149, 2294720	147	266	7207	0.55	1.00	1.00	100.00
	SMART	SM00355:ZnF_C2H2	3	1.73	0.99	2293303, 2299149, 2294720	74	266	3277	0.50	1.00	1.00	100.00
Annotation Cluster 15	GOTERM_MF_FAT	GO:0005524~ATP binding	5	2.89	1.00	2291072, 2299418, 2297082, 2295025, 2299106	78	629	4047	0.41	1.00	1.00	100.00
Enrichment Score: 0.01	GOTERM_MF_FAT	GO:0032559~adenyl ribonucleotide binding	5	2.89	1.00	2291072, 2299418, 2297082, 2295025, 2299106	78	630	4047	0.41	1.00	1.00	100.00
	GOTERM_MF_FAT	GO:0030554~adenyl nucleotide binding	5	2.89	1.00	2291072, 2299418, 2297082, 2295025, 2299106	78	660	4047	0.39	1.00	1.00	100.00
	GOTERM_MF_FAT	GO:0001883~purine nucleoside binding	5	2.89	1.00	2291072, 2299418, 2297082, 2295025, 2299106	78	660	4047	0.39	1.00	1.00	100.00
	GOTERM_MF_FAT	GO:0001882~nucleoside binding	5	2.89	1.00	2291072, 2299418, 2297082, 2295025, 2299106	78	664	4047	0.39	1.00	1.00	100.00
	GOTERM_MF_FAT	GO:0032553~ribonucleotide binding	6	3.47	1.00	2291072, 2299418, 2297082, 2295151, 2295025, 2299106	78	770	4047	0.40	1.00	1.00	100.00
	GOTERM_MF_FAT	GO:0032555~purine ribonucleotide binding	6	3.47	1.00	2291072, 2299418, 2297082, 2295151, 2295025, 2299106	78	770	4047	0.40	1.00	1.00	100.00
	GOTERM_MF_FAT	GO:0017076~purine nucleotide binding	6	3.47	1.00	2291072, 2299418, 2297082, 2295151, 2295025, 2299106	78	806	4047	0.39	1.00	1.00	100.00
	GOTERM_MF_FAT	GO:0000166~nucleotide binding	7	4.05	1.00	2291072, 2299418, 2300269, 2297082, 2295151, 2295025, 2299106	78	963	4047	0.38	1.00	1.00	100.00

36-h B. a.

Annotation Cluster 1	SP_PIR_KEYWORDS	transmembrane	7	10.45	0.16	2296588, 2298218, 2293165, 2295322, 2291073, 2289525, 2291961	25	38	240	1.77	1.00	1.00	77.52
Enrichment Score: 0.63	SP_PIR_KEYWORDS	membrane	6	8.96	0.16	2296588, 2298218, 2295322, 2291073, 2289525, 2291961	25	30	240	1.92	1.00	0.95	78.72
	SP_PIR_KEYWORDS	transport	4	5.97	0.21	2296588, 2293165, 2291073, 2289525	25	16	240	2.40	1.00	0.92	86.64
	GOTERM_CC_FAT	GO:0016021~integral to membrane	9	13.43	0.27	2296588, 2298218, 2300676, 2293165, 2295322, 2291073, 2289525, 2291961, 2298754	12	59	99	1.26	0.98	0.98	87.97
	GOTERM_CC_FAT	GO:0031224~intrinsic to membrane	9	13.43	0.27	2296588, 2298218, 2300676, 2293165, 2295322, 2291073, 2289525, 2291961, 2298754	12	59	99	1.26	0.98	0.98	87.97
	GOTERM_BP_FAT	GO:0006811~ion transport	3	4.48	0.40	2296588, 2298218, 2289525	15	19	198	2.08	1.00	1.00	99.42
Annotation Cluster 2	GOTERM_MF_FAT	GO:0000166~nucleotide binding	6	8.96	0.46	2299418, 2297623, 2298218, 2298573, 2290523, 2289458	25	51	279	1.31	1.00	1.00	99.85
Enrichment Score: 0.25	GOTERM_MF_FAT	GO:0032553~ribonucleotide binding	5	7.46	0.48	2299418, 2297623, 2298218, 2290523, 2289458	25	41	279	1.36	1.00	1.00	99.91
	GOTERM_MF_FAT	GO:0032555~purine ribonucleotide binding	5	7.46	0.48	2299418, 2297623, 2298218, 2290523, 2289458	25	41	279	1.36	1.00	1.00	99.91
	GOTERM_MF_FAT	GO:0017076~purine nucleotide binding	5	7.46	0.50	2299418, 2297623, 2298218, 2290523, 2289458	25	42	279	1.33	1.00	1.00	99.94
	GOTERM_MF_FAT	GO:0005524~ATP binding	4	5.97	0.62	2299418, 2298218, 2290523, 2289458	25	36	279	1.24	1.00	1.00	100.00
	GOTERM_MF_FAT	GO:0032559~adenyl ribonucleotide binding	4	5.97	0.62	2299418, 2298218, 2290523, 2289458	25	36	279	1.24	1.00	1.00	100.00
	GOTERM_MF_FAT	GO:0001882~nucleoside binding	4	5.97	0.64	2299418, 2298218, 2290523, 2289458	25	37	279	1.21	1.00	1.00	100.00
	GOTERM_MF_FAT	GO:0001883~purine nucleoside binding	4	5.97	0.64	2299418, 2298218, 2290523, 2289458	25	37	279	1.21	1.00	1.00	100.00
	GOTERM_MF_FAT	GO:0030554~adenyl nucleotide binding	4	5.97	0.64	2299418, 2298218, 2290523, 2289458	25	37	279	1.21	1.00	1.00	100.00
Annotation Cluster 3	GOTERM_MF_FAT	GO:0046914~transition metal ion binding	5	7.46	0.67	2297343, 2293303, 2292414, 2296172, 2293642	25	51	279	1.09	1.00	1.00	100.00
Enrichment Score: 0.09	GOTERM_MF_FAT	GO:0008270~zinc ion binding	4	5.97	0.75	2297343, 2293303, 2292414, 2293642	25	43	279	1.04	1.00	1.00	100.00
	GOTERM_MF_FAT	GO:0046872~metal ion binding	5	7.46	0.87	2297343, 2293303, 2292414, 2296172, 2293642	25	67	279	0.83	1.00	1.00	100.00
	GOTERM_MF_FAT	GO:0043167~ion binding	5	7.46	0.90	2297343, 2293303, 2292414, 2296172, 2293642	25	71	279	0.79	1.00	1.00	100.00
	GOTERM_MF_FAT	GO:0043169~cation binding	5	7.46	0.90	2297343, 2293303, 2292414, 2296172, 2293642	25	71	279	0.79	1.00	1.00	100.00

24-h C+A vs C-A

Annotation Cluster 1	INTERPRO	IPR008962:PapD-like	2	12.50	0.03	2297118, 2290424	12	17	7207	70.66	0.44	0.44	18.41
	INTERPRO	IPR000535:Major sperm protein	2	12.50	0.04	2297118, 2290424	12	24	7207	50.05	0.55	0.33	24.98
	SP_PIR_KEYWORDS	cytoskeleton	2	12.50	0.06	2297118, 2290424	8	28	3152	28.14	0.61	0.61	35.25
	GOTERM_CC_FAT	GO:0043228~non-membrane-bounded organelle	3	18.75	0.07	2292671, 2297118, 2290424	3	347	1317	3.80	0.44	0.44	32.50
	GOTERM_CC_FAT	GO:0043232~intracellular non-membrane-bounded organelle	3	18.75	0.07	2292671, 2297118, 2290424	3	347	1317	3.80	0.44	0.44	32.50
	GOTERM_BP_FAT	GO:0006793~phosphorus metabolic process	3	18.75	0.09	2299155, 2290666, 2290605	5	390	2832	4.36	0.86	0.86	52.76
	GOTERM_BP_FAT	GO:0006796~phosphate metabolic process	3	18.75	0.09	2299155, 2290666, 2290605	5	390	2832	4.36	0.86	0.86	52.76

36-h C+A vs C-A

Annotation Cluster 1	INTERPRO	IPR000742:EGF-like, type 3	3	6.98	0.02	A8QH07, A8PIP1, A8PED3	32	56	7207	12.07	0.88	0.88	23.07
Enrichment Score: 1.46	SMART	SM00181:EGF	3	6.98	0.03	A8QH07, A8PIP1, A8PED3	14	69	3277	10.18	0.46	0.46	20.54
	INTERPRO	IPR006210:EGF-like	3	6.98	0.04	A8QH07, A8PIP1, A8PED3	32	69	7207	9.79	0.96	0.80	32.18
	SP_PIR_KEYWORDS	egf-like domain	3	6.98	0.04	A8QH07, A8PIP1, A8PED3	18	57	3152	9.22	0.70	0.70	27.68
	INTERPRO	IPR013032:EGF-like region, conserved site	3	6.98	0.06	A8QH07, A8PIP1, A8PED3	32	92	7207	7.34	1.00	0.84	48.33
Annotation Cluster 2	GOTERM_MF_FAT	GO:0043167~ion binding	4	9.30	0.83	A8QH07, A8NF63, A8PIP1, A8Q8M9	17	1050	4047	0.91	1.00	1.00	100.00
Enrichment Score: 0.06	GOTERM_MF_FAT	GO:0043169~cation binding	4	9.30	0.83	A8QH07, A8NF63, A8PIP1, A8Q8M9	17	1050	4047	0.91	1.00	1.00	100.00
	GOTERM_MF_FAT	GO:0046872~metal ion binding	3	6.98	0.94	A8QH07, A8PIP1, A8Q8M9	17	1032	4047	0.69	1.00	1.00	100.00
