# Supplementary material for: Characterization of innate immunity genes in the parasitic nematode Brugia malayi
Source: Symbiosis. 2016 Jan 5;68:145–55. doi: 10.1007/s13199-015-0374-7 (PMC4826884; doi:10.1007/s13199-015-0374-7)
Supplement: Supplementary file 3 — (PDF 216 kb) [file 13199_2015_374_MOESM3_ESM.pdf]

Electronic Supplementary Material 3

Journal: Symbiosis

Characterization of innate immune genes in the parasitic nematode *Brugia malayi*

Silvia Libro[1], Barton E. Slatko, Jeremy M. Foster

New England Biolabs, Inc., Genome Biology Division, 240 County Road, Ipswich, MA USA 01938

[1] Corresponding author: libro@neb.com - Ph: 978-380-7311

Supplementary Results and Discussion. Additional immune-related transcripts and stress response genes that underwent significant differential expression in treated worms compared to controls. Transcripts expression levels are indicated as log2 fold change of Fragments Per Kilobase of transcript per Million mapped reads (FPKM)-normalized count data.

Putative allergens and modulators of the human host`s immunity

- Nematode polyprotein allergen related Npa-1 (0.9 in 24-h *B. a.*; 1.1 in 22-h *E. c.*)
- Cysteine-rich secretory protein/allergen V5/Tpx-1-related (0.9 in 24-h *B. a.*; 1.0 in 24-h dRNA; 1.6 in 22-h *E. c.*)
- Chromadorea abundant larval transcript (ALT) family member (1.2 in 22-h *E. c.*)
- Macrophage migration inhibitory factor (Mif-1) (1.1 in 22-h *E. c.*)
- Interleukin-17 family member (1.2 in 22-h *E. c.*)

Antioxidants and xenobiotic detoxicants

- Sestrin-1 (-1.1 in 22-h *E. c.;* -1.3 in 24-h *B. a.*)
- Thioredoxin (n = 2, 0.7 and 0.9)
- Glutathione S-transferase (n = 2, 0.7 and inf – i.e., not expressed in the control – in 22-h *E. c.*)
- Glutathione peroxidase (1.3 in 22-h *E. c.;* -1.4 in 36-h dsRNA)
- Major facilitator superfamily members (n = 7, 0.9 - 1.7 in 22-h *E. c.*; n=2, both 1.0 in 24-h *B. a.*; 1.0 in 20-h *E. c.;* 1.1 in 12-h dsDNA; 0.9 in 16-h dsDNA; -0.8 in 36-h dsRNA)
- Tetracycline resistance protein TetA (0.9 in 22-h *E. c.*)
- Multidrug-R protein (0.8 in 22-h *E. c.*)

- ABC transporter type 1 (1.3 in 22-h *E. c.*)

Stress-response genes

- Alpha crystallin/Heat shock proteins (n = 2, 2.1 and 5.7 in 22-h *E. c;* 3.1 and -3.2 in 36-h dsRNA; 4 and -5 in 36-h *B. a.*)
- Heat shock protein DnaJ (-0.9 in 22-h *E. c.;* -1.0 in 24-h *B. a.*)
- Hsp20-like chaperone (1.0 in 22-h *E. c.*)
- JNK1-Associated Membrane Protein (0.9 in 22-h *E. c.*)
- Hyl-2 ceramide synthase (1.1 in 24-h dsRNA)
